# Anle138b mitigates post-hypoxic cognitive impairment, α-Synuclein aggregation and UPR activation in *Drosophila melanogaster*

**DOI:** 10.1186/s40478-025-02099-5

**Published:** 2025-11-11

**Authors:** Aaron T. Fehr, Jennifer Jung, Alma Kokott-Vuong, Sabri E. M. Sahnoun, Aya A. Ezzat, Michael Huber, Tonya M. Bliss, Aaron Voigt, Jörg B. Schulz, Pardes Habib

**Affiliations:** 1 Department of Neurology, Medical Faculty, RWTH Aachen University, 52074 Aachen, Germany; 2 Department of Nuclear Medicine, University Hospital Aachen, RWTH Aachen University, 52074 Aachen, Germany; 3 Institute of Biochemistry and Molecular Immunology, Medical Faculty, RWTH Aachen University, 52074 Aachen, Germany; 4 Department of Neurosurgery and Stanford Stroke Center, Stanford University School of Medicine, Stanford, CA USA; 5 JARA-BRAIN Institute Molecular Neuroscience and Neuroimaging, Forschungszentrum Jülich GmbH and RWTH Aachen University, 52074 Aachen, Germany

**Keywords:** Post-stroke cognitive impairment, Neurodegeneration, Stroke, Hypoxia, Neuroprotective therapy, ER stress

## Abstract

Cerebral ischemia increases the risk of post-stroke cognitive impairment (PSCI), but the underlying molecular mechanisms remain unclear. Emerging evidence suggests that hypoxia/ischemia-induced oxidative and endoplasmic reticulum (ER) stress may contribute to protein misfolding and α-Synuclein (α-Syn) aggregation, potentially triggering the unfolded protein response (UPR) to alleviate ER stress. Using bimolecular fluorescence complementation in *Drosophila melanogaster* and HEK-293 cells, we investigated the effect of acute, repetitive and chronic hypoxia on α-Syn aggregation, UPR activation, mortality, longevity, locomotor function, sleep, and cognition. Furthermore, we evaluated the post-hypoxic in vivo biodistribution and therapeutic efficacy of the aggregation inhibitor anle138b. Acute severe hypoxia induced more α-Syn aggregation than chronic or repetitive hypoxia, resulting in higher mortality, reduced longevity, delayed motor recovery, cognitive impairment, and activation of the detrimental PERK branch of the UPR. Anle138b significantly reduced α-Syn aggregation, repressing post-hypoxic PERK activation and improving survival and decision-making. Our findings demonstrate the effectiveness of anle138b in mitigating hypoxia-induced α-Syn aggregation and cognitive impairment, paving the way for future studies on its potential as a therapeutic strategy for PSCI.

**Graphical abstract:**

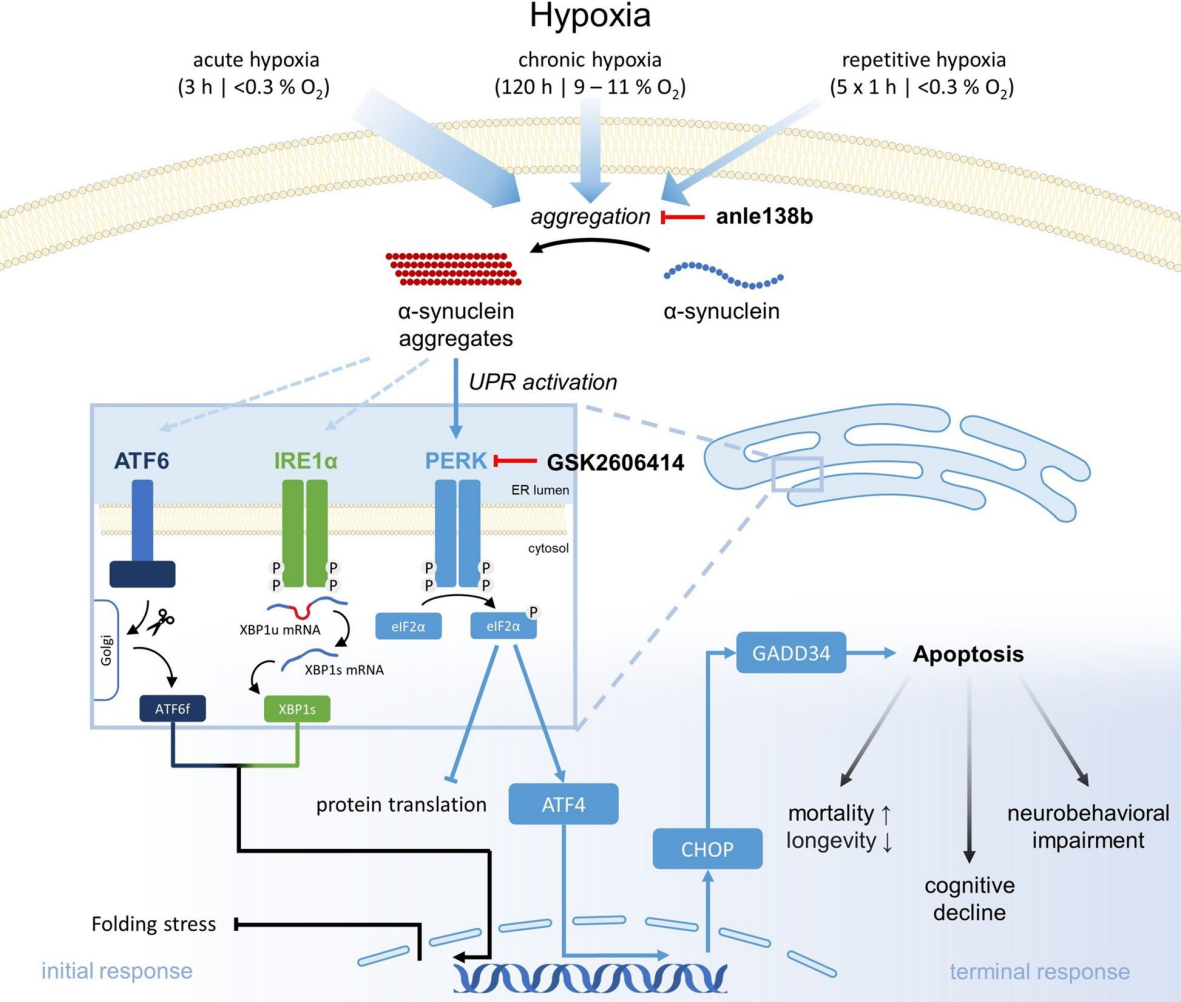

## Introduction

Hypoxia is a crucial condition underlying numerous diseases such as ischemic heart failure, pulmonary hypertension, and cerebral ischemia [1, 2]. The latter is one of the leading causes of death and disability in adulthood worldwide [3, 4]. Although advances in acute and secondary care of stroke patients have significantly increased post-stroke survival rates over the past decades, burdens such as chronic motor deficits and post-stroke cognitive impairment (PSCI) remain a major challenge [5–7].

PSCI describes a heterogeneous group of cognitive impairment symptoms, which typically affect complex cognitive functions, including orientation, memory, visuospatial function, executive function and attention [8–10]. Over 50% of all stroke patients experience PSCI within the first 1.5 years post-stroke, and between 6 and 30% of the patients even meet the criteria for post-stroke dementia [11–13].

Initially, it was assumed that similar pathophysiological mechanisms to those in vascular dementias caused PSCI. However, more recent studies have reported that multifactorial and more Alzheimer’s Disease-related mechanisms contribute to approximately 30% of all cases of post-stroke dementia [12]. Numerous neurodegenerative diseases caused by aggrego-/synucleinopathies including Alzheimer’s disease, Lewy-body dementia and Parkinson’s Disease share common key pathological mechanisms with hypoxia [9, 14–17]. Cerebral hypoxia is known to induce oxidative stress, mitochondrial impairment, endoplasmic reticulum (ER)-stress, autophagy imbalance and neuroinflammation as well as α-Synuclein (α-Syn) aggregation, all of which are implicated in the pathogenesis of these neurodegenerative diseases [5, 14, 18–21].

α-Syn is a small, soluble 140 amino acid protein found primarily in the presynaptic terminals of neurons in the brain [17]. Its exact physiological function is still not fully understood, but it is believed to play a role in synaptic function, cell membrane interactions, vesicle transport and dopamine synthesis in the central nervous system [17, 22, 23]. α-Syn is predominantly found in either an unfolded or an alpha-helical structural conformation [24]. However, various factors such as genetic mutations, phosphorylation, environmental stress and interactions with pathological chaperone proteins can promote misfolding of α-Syn and induce its conversion to a beta-sheet conformation [22, 24, 25]. In this misfolded beta-sheet form, α-Syn is more likely to form protein oligomers, fibrils and aggregates, leading to the accumulation of intracellular inclusions in the cerebral cortex and hippocampus, accompanied by ER-stress and neurological deficits [22, 24].

A promising pharmacological approach to mitigate α-Syn aggregation is the use of aggregation inhibitors, such as anle138b. Anle138b is a small molecule inhibitor that was developed through a systematic high-throughput screening process and medicinal chemistry optimization [26]. A recent study using NMR spectroscopy demonstrated that anle138b binds a cavity within lipidic α-Syn fibrils, which effectively impedes the spontaneous formation of beta-sheet structures and inhibits the maturation of α-Syn aggregates [26–28]. Anle138b demonstrated beneficial effects in several in vitro and in vivo studies and prevented disease progression in PD and multiple system atrophy mouse models by blocking the formation and accumulation of α-Syn oligomers in the brain [26, 29–33]. The favorable pharmacokinetic profile and lack of toxicity at therapeutic doses currently allow to proceed into human clinical trials [26, 34].

While anle138b pharmacologically targets α-Syn aggregation, the unfolded protein response (UPR) is a prominent cellular pathway responding upon accumulation of un-/misfolded proteins like α-Syn in the ER [35, 36]. It consists of three transmembrane sensors: inositol-requiring enzyme 1α (IRE1α), protein kinase RNA-like endoplasmic reticulum kinase (PERK) and activating transcription factor 6 (ATF6) [37]. Under physiological conditions, the ER chaperone glucose-regulated protein 78 (GRP78) binds to the three transmembrane sensors. However, upon accumulation of unfolded or misfolded proteins, GRP78 dissociates from the transmembrane proteins, leading to the activation of a series of cascades to cope with the folding stress [37–39]. The IRE1α and ATF6 branch promote the transcription of genes encoding for proteins involved in protein folding, ER-associated degradation (ERAD), protein quality control, autophagy, phospholipid synthesis and mRNA decay by regulated IRE1-dependent decay (RIDD) [37]. PERK phosphorylates the eukaryotic translation initiation factor 2α (eIF2α), leading to a global reduction in protein synthesis to decrease the load of protein folding in the ER and mitigate the production of potentially harmful misfolded proteins [37, 40]. However, upon prolonged and severe ER-stress, the PERK branch activates activating transcription factor 4 (ATF4), which upregulates the proapoptotic transcription factor C/EBP-homologous protein (CHOP) or Xrp1 in *Drosophila melanogaster*. CHOP (or Xrp1) induces the expression of growth arrest and DNA damage-inducible 34 (GADD34), which triggers a series of mechanisms that culminate in the induction of apoptosis [35, 37, 40, 42, 43]. Thus, the PERK branch plays a dual role in the UPR, both assisting the protein folding process and reducing protein synthesis, but also promoting cell death as a last resort.

Despite successful cerebral reperfusion achieved by thrombectomy or thrombolysis, stroke patients frequently exhibit increased rates of dementia, raising the question of which type of hypoxic stimulus (acute, repetitive, or chronic) leads to the most pronounced α-Syn aggregation. Moreover, the subsequent impact of hypoxia-induced α-Syn aggregation on neurobehavioral dysfunction and UPR activation as well as the potential of aggregation inhibitors like anle138b to mitigate these post-hypoxic damages remain unclear.

In this study, we established a reliable protocol to induce acute, repetitive, and chronic hypoxia and used a Bimolecular fluorescence Complementation (BiFC) model to detect α-Syn aggregation in *Drosophila melanogaster* and HEK-293 cells. *Drosophila melanogaster* is a widely used model organism due to its short lifecycle, high degree of genetic conservation, ease of genetic manipulation, and its ability to yield high-throughput results in vivo [18, 44, 45]. We assessed post-hypoxic mortality rates, and analyzed neurobehavioral outcomes on multiple levels, including motor function, activity levels, sleep pattern, longevity and cognition. We evaluated expression levels of UPR markers upon inhibition of PERK and/or α-Syn aggregation to explore a mechanistic link. For PERK inhibition, we applied GSK2606414 (GSK), a small molecule that selectively inhibits the activation of the PERK branch via binding to the PERK cytosolic kinase domain in the ATP-binding site cleft [46–48]. Furthermore, we utilized a modified radiolabeling protocol to assess the biodistribution of the aggregation inhibitor anle138b and evaluated its therapeutic effects in *Drosophila melanogaster*.

We provide evidence that acute hypoxia causes higher α-Syn aggregation levels than chronic and repetitive hypoxia, leading to increased mortality rates, reduced life expectancy, longer recovery times, impaired cognitive function and upregulation of the proapoptotic PERK/Xrp1/GADD34 branch of the UPR. The activation of the PERK branch seems to be triggered by α-Syn aggregation, whereas inhibiting PERK fails to reduce aggregation levels. The aggregation inhibitor anle138b mitigates these effects by effectively reducing α-Syn aggregation and downregulating the PERK branch, resulting in higher life expectancy and improved cognitive function*.* Our findings highlight the ability of anle138b to mitigate hypoxia-induced α-Syn aggregation and its detrimental effects, encouraging further research to explore its potential role in PSCI treatment.

## Materials and methods

### Drosophila melanogaster

*Drosophila melanogaster* P{w[+ mW.hs] = GawB}elav[C155] (BL458) were obtained from the Bloomington Drosophila Stock Centre (Bloomington, IN, USA) and used as control flies containing no α-Syn (control flies in text). After crossing P{w[+ mW.hs] = GawB}elav[C155]; PBac{attB[+ mC] = UAS-Hsap\SNCA:VN}/CyO with w[*]/Y; P{w[ +] = UAS-Hsap\[SNCA:VC}/CyO, we used 1 to 5 days old male flies from F1 P{w[+ mW.hs] = GawB}elav[C155]/Y; P{w[ +] = UAS-Hsap\SNCA:VC}/P{w[ +] = UAS-Hsap\SNCA:VN} (VN/VC flies in text) as well as P{w[+ mW.hs] = GawB}elav[C155]/Y; PBac{attB[+ mC] = UAS-Hsap\SNCA:VN}/CyO and P{w[+ mW.hs] = GawB}elav[C155]/Y; P{w[ +] = UAS-Hsap\SNCA:VC}/CyO (Curly flies in text) for conducting the experiments.

While VN/VC flies express the full BiFC construct, enabling a quantitative analysis of the α-Syn aggregation levels, Curly flies do not contain the full construct, but still can express α-Syn.

The flies were kept in plastic vials with standard cornmeal food at 23 °C under a 12 h / 12 h light/dark cycle [45]. Flies were relocated to new vials with fresh food every 5 days.

### Anle138b and GSK2606414 treatment

100 µl of anle138b (250 µM) (Sigma-Aldrich #SML1515, Taufkirchen, Germany) and/or GSK2606414 (10 µM) (Calbiochem #516535, Darmstadt, Germany) were placed on top of the cornmeal food and evenly dispersed. Food covered with 100 µl of Dimethyl sulfoxide (DMSO, #67-68-5, Merck KGaA, Darmstadt, Germany) served as vehicle control. Food vials were left to dry for 48 h to allow the DMSO to evaporate. The flies were transferred to the vials containing the inhibitors immediately after hatching and kept on the food until the hypoxia for 1–5 days.

### Hypoxia paradigms in *Drosophila melanogaster*

We established a reliable protocol for acute, repetitive and chronic hypoxia in *Drosophila melanogaster*. To induce acute hypoxia, we exposed flies to 3 h of severe hypoxia (O_2_ < 0.3%). For repetitive hypoxia, the flies were subjected to 1 h of severe hypoxia (O_2_ < 0.3%) every day for 5 days, with intermittent reoxygenation periods. Chronic hypoxia was induced by subjecting the flies to 120 h of mild hypoxia (O_2_ between 9 and 11%).

### Hypoxia induction in *Drosophila melanogaster*

Hypoxia was induced using nitrogen (N_2_) in a previously described self-constructed hypoxia chamber [18, 44, 45]. Oxygen levels, humidity, temperature and air pressure were monitored throughout every hypoxia and normoxia to enable comparability and reproducible results. Before hypoxia, the flies were transferred to empty vials without food, with every vial containing 20 flies. The vials were then sealed with gauze to allow efficient gas exchange between the hypoxia chamber and the vial and placed into the hypoxia chamber. Then, the hypoxia chamber was flooded with N_2_ until oxygen levels below 0.3% for acute and repetitive hypoxia or between 9 and 11% for chronic hypoxia were reached. After hypoxia, the hypoxia chamber was flooded with room air and the flies were transferred back to vials with food for reoxygenation or immediately used for analysis. During reoxygenation, we monitored temperature, air pressure and humidity to guarantee constant environmental conditions. All subsequent experiments were performed 3 times with 3 technical replicates of 20 flies per genotype and condition (total of 180 flies per genotype and condition).

### Bimolecular fluorescence complementation (BiFC) aggregation assessment

#### Preparation of fly head lysates

For aggregation analysis, the flies were transferred to 1.5 ml Eppendorf tubes and snap-frozen in liquid nitrogen. The fly heads were then separated from the body and subsequently lysed in Radioimmunoprecipitation assay (RIPA) buffer (#11873580001, Roche Basel, Switzerland) (10 µl per fly head). A speed mill (Analytic Jena, Jena, Germany) was used to homogenize the fly heads.

#### Protein concentration assessment

The protein concentration of the fly head lysates was assessed with the DC Protein Assay Kit (Bio-Rad, Hercules, CA, USA) following the manufacturer’s instructions and measured with a plate reader (Infinite 200 Pro Reader, Tecan, Männedorf, Switzerland).

#### Venus signal measurement

The fly head lysates were diluted to a final concentration of 1 mg/ml total protein. 5 µl of each sample was mixed with 45 µl of ultrapure H_2_O in a white 96-well plate. VENUS fluorescence levels were measured in duplicates at 529–530 nm after excitation at 485 nm in a plate reader (Infinite 200 Pro Reader, Tecan, Männedorf, Switzerland).

### Thioflavin T assay

Fly head protein lysates were obtained as described above. Thioflavin T (T3516, Sigma-Aldrich, Darmstadt, Germany) was diluted in phosphate-buffered saline (PBS) to create a 20 µM working solution.

90 µl of protein samples was incubated with 10 µl Thioflavin T working solution. Thioflavin fluorescence levels were measured in duplicates at 485 nm after excitation at 450 nm in a plate reader (Infinite 200 Pro Reader, Tecan, Männedorf, Switzerland).

### ATP assay

ATP assay was performed with the Roche ATP Bioluminescence Assay Kit HS II (Cat. No. 11 699 709 001, Roche, Darmstadt, Germany), according to official protocol.

In short, the ATP standard was diluted by serial dilution.

Fly heads were collected in the lysis buffer supplied with the assay kit. Fly head lysis was performed in a speedmill (8 min), as described above. Samples were heated to 95 °C for 2 min and centrifuged with 20,000 g at 4 °C for 1 min.

187.5 µl of Dilution Buffer, 2.5 µl of clear supernatant of the samples or ATP standards and 10 µl luciferase were added to each well of a 96-well plate.

Luminescence was directly measured at 562 nm, repeated measurements were performed. ATP concentrations were calculated based on the standard curve.

### Mortality rate

For mortality rate assessment, 1 to 5 days old male VN/VC and control flies were subjected to 2 to 6 h of severe hypoxia (< 0.3% O_2_) and the mortality rate was determined after 6, 24, 48, 72, 96 and 120 h of reoxygenation. A non-linear regression analysis of the mortality rates after 120 h of reoxygenation was used in Figs. 3f and 10f.

### Longevity assay

For longevity assessment, 1 to 5 days old male VN/VC and control flies (Fig. 3g–k) or VN/VC flies treated with DMSO or anle138b (Fig. 6h–i) were subjected to 3 h of severe hypoxia (< 0.3% O_2_). The survival rate was assessed daily until all flies had died. During this period, the flies were transferred to new vials with fresh food every 2 days to minimize the impact of exogenous factors such as fungus formation or variations in food quality, thus ensuring reliable results.

### Drosophila activity monitoring (DAM) assay

A Drosophila Activity Monitoring (DAM) System (Model DAM2, Trikinetics Inc., Waltham, MA, USA) was used to assess the individual activity of the flies, as described before [45]. After hypoxia/normoxia, the flies were individually transferred into the DAM tubes, containing food (2% agar, 4% sucrose). The number of light beam interruptions, representing the flies’ activity, was monitored in each DAM tube individually every 10 min over an observation period of 5 days with a 12 h/12 h light/dark cycle. The DAMSystem3 Software (Trikinetics Inc., Waltham, MA, USA) was used for data acquisition. Data were organized in the DAMFileScan Software (Trikinetics Inc., Waltham, MA, USA). The experiment was repeated 3 times in total, including 32 flies for each genotype and treatment (total of 96 flies per genotype and treatment).

### Value-based feeding decision (VBFD) assay

For the assessment of the cognitive abilities and the decision-making ability of the flies, a Value Based Feeding Decision (VBFD) assay was performed 10 days after hypoxia/normoxia [49]. Therefore, the flies were food-deprived on starving food (1% agar) 12 h prior to the VBFD. After 12 h of starvation, groups of 20 flies were placed on a petri dish containing 4 droplets (20 µL) of both a sucrose solution (150 mM) (D(+)-Saccharose, #4621.1, Carl Roth GmbH, Karlsruhe, Germany) as well as an arabinose solution (150 mM) (D-(−)-arabinose, #A10357, ThermoFisher GmbH, Kandel, Germany). Both sugar solutions were color labeled with glucose-free food coloring (ultramarine blue, bright red, Wenburg, Cologne, Germany) prior to the assay. After 3 h, the feeding decision was assessed under a microscope by observation of the abdomen color (red: sucrose, blue: arabinose, violet: both or no color: non-eater). As flies can metabolize sucrose but not arabinose, a food choice in favor of the sucrose solution is to be expected. Fisher’s exact test was used to compare the group of flies which chose only sucrose and the group which had consumed arabinose or no sugar at all.

### HEK-293 cell culture

For in vitro experiments, human embryonic kidney 293T (HEK-293) cells (HEK293T; Leibniz Institute DSMZ-German Collection of Microorganisms and Cell Cultures GmbH, Braunschweig, Germany) were cultured in Dulbecco’s Modified Eagle Medium (DMEM; PAN-Biotech, Aidenbach, Germany) supplemented with 10% fetal bovine serum (FBS; PAN-Biotech, Aidenbach, Germany) and 0.5% Penicillin/Streptomycin (PAN-Biotech; Aidenbach, Germany) at an incubator temperature of 37 °C.

### Transfection

The transfection of HEK-293 cells was performed as previously described [50]. In short, HEK-293 cells were seeded with a density of 1.2 × 10^4^ cells / cm^2^. After 24 h, the medium was replaced with fresh DMEM and the cells were transfected with 1.5 µg / well of both DNA constructs (VN-α-Syn and VC-α-Syn) using Metafectene (Biontex Laboratories, Munich/Laim, Germany) following the manufacturer’s instructions.

### Hypoxia induction in HEK-293 cells

HEK-293 cells were subjected to 3 h of acute hypoxia 24 h after transient transfection as previously described [51–54]. The hypoxia was conducted in our customized hypoxia chamber (#C174, C21, BioSpherix, Parish, NY, United States) by flooding the chamber with N_2_. The hypoxia was performed at oxygen concentrations below 0.1%. Oxygen levels, temperature, and air pressure were monitored throughout the whole hypoxia to allow stable hypoxia conditions. The hypoxia chamber was placed in a humidified incubator at 37 °C on an orbital shaker (30 rpm) to enhance O_2_ depletion in the medium. Normoxia controls were kept in the same incubator at 5% CO_2_.

### Treatment in HEK-293 cells

After hypoxia, anle138b (Sigma-Aldrich #SML1515, Taufkirchen, Germany) diluted in DMSO (10 µM concentration) or pure DMSO (DMSO, #67-68-5, Merck KGaA, Darmstadt, Germany) were added to each well with HEK-293 cells. Treatment was left on the cells during a reoxygenation period of 24 h.

### Flow cytometry

After 0, 6 and 24 h of reoxygenation, the α-Syn aggregation levels were assessed by flow cytometry. Therefore, the HEK-293 cells were transferred to 1.5 ml Eppendorf tubes by washing with PBS followed by trypsinization (Trypsin/EDTA, PAN-Biotech, Aidenbach, Germany) and centrifugation in cold RPMI supplemented with 5% FBS for 5 min at 400×*g*.

The HEK-293 cells were washed twice with flow cytometry buffer (2% FBS, 2 mM EDTA in PBS) and eventually resolved in 200 µl of flow cytometry buffer. Subsequently, the cell dilution was transferred to flow cytometry tubes and fixated with 200 µl of paraformaldehyde (4%, PFA). A FACSCantoTM II (BD Transduction Laboratories, Franklin Lakes, NJ, USA) was used for flow cytometry analysis. Viable cells were gated based on forward and side scatter (FSC/SSC) characteristics to exclude debris and dead cells. An empty plasmid control was used to exclude background, and a GFP-transfected positive control served as a template for definition of low (10^2^–10^3^), medium (10^3^–10^4^) and high (> 10^4^) intensity fluorescence. 50,000 cells per sample were acquired. Signal amplification was set to keep background fluorescence below 100. The FlowJo software (Treestar Inc., Ashland, OR, USA) was used for data analysis.

### Fluorescence microscopy

For Fluorescence microscopy, cells were seeded to Poly-L-lysine covered coverslips. After hypoxia and reoxygenation, coverslips were washed with phosphate-buffered saline (PBS), fixed for 30 min with 4% formaldehyde in PBS and permeabilized with 0.2% Triton X-100. Subsequently, the cover slips were washed with PBS for 4 times before Thioflavin S staining.

#### Thioflavin S staining

Thioflavin S (T1892, Sigma-Aldrich, Darmstadt, Germany) staining was performed according to an established protocol (Steps 51–56) [55]. In short, the cover slips were incubated with the 0.05% Thioflavin S solution (dissolved in 50% ethanol/water) for 15 min at 20–22 °C in the dark. Subsequently, cover slips were washed twice with 50% ethanol and once with 80% ethanol, each washing step for 20 min. Finally, cover slips were washed with PBS once before mounting.

#### Imaging

Stainings were evaluated using a Echo RON-K fluorescence microscope (Echo, San Diego, CA, USA). Picture analysis was performed using ImageJ. Fluorescence was compared based on the mean fluorescence per cell.

### RT-qPCR

Gene expression levels were assessed as previously reported [18, 44]. Briefly, fly heads were homogenized in TRIzol Reagent (#15596026, Invitrogen, Darmstadt, Germany) with a speed mill (Analytic Jena, Jena, Germany). After addition of Chloroform, the samples were incubated on ice for 10 min and subsequently centrifuged for 20 min (11.4 rpm, 4 °C). Afterwards, the upper aqueous phase was transferred into a fresh 1.5 ml Eppendorf tube and incubated with Isopropanol overnight at − 20 °C. On the next day, the samples were centrifuged for 60 min (11.4 rpm, 4 °C) and the supernatant was carefully discarded. The pellet was washed 3 times with ice cold ethanol (75%) and then resolved in 15 µl of ultrapure H_2_O. The samples were diluted to a concentration of 1 µg/15 µl. The iScript cDNA Synthesis Kit (BioRad Laboratories, Hercules, CA, USA) was used for synthesizing complementary DNA. RNAse free H_2_O (Merck, 64293, Darmstadt, Germany) functioned as a no template control (NTC). RT-qPCR analysis was conducted with the MyIQ RT-qPCR detection system (Bio-Rad, Munich, Germany). The target genes and housekeeping genes were assessed as cycle threshold (Ct values) and the qbase + software (Biogazelle, Gent, Belgium) was used for relative quantification with the ΔΔCt method. Data are expressed as the fold change compared to the housekeeping gene. The following primers (5′→3′) were used:

**Table:** 

Gene	Forward primer	Reverse primer
*eEF1α2*	GCGTGGGTTTGTGATCAGTT	GATCTTCTCCTTGCCCATCC
*Actin5c*	TTTCAAACCGTGCGGTCGCT	CATCACACCCTGGTGACGGG
*Grp78*	TCTTGTACACACCAACGCAGG	CAAGGAGCTGGGCACAGTGA
*Edem1*	GAAGCAGTATTCCAAGGCAAGA	GGCGCAGGTAACCATCGTAG
*Atf4*	AGGCCATAGTACCCGCAAAC	CCGCCTGTTTGTAAGCATCG
*Gadd34*	CGAGCAATATCGGTTCGGG	TGATGCACCTTGTTTGGCTTC
*Xbp1s*	CTCGAGTTCGGGATACGCAT	CCAGGTTAGATGGTCCAGGC
*Manf*	AGATCGAAACGGCCTTCAAAA	GTGGCGGATTCTTCCAGACC
*dPerk*	CAGGAGCCATCGAGAGTGAT	AACGGTGACGTGTTGACCTC
*eIF2α*	ATCAACCTGATAGCACCGCC	CTCCATCGTACTCGCTGGTC
*Xrp1*	GCAGCCGTTCAAAACGTAGT	TGCGATCTCTGAGTCAACCG
*Diap1*	ATGTCGAATCTCGGCCCGTA	GCGCTCAAAGCGAGTTGTC
*Debcl*	CCCAATCCCTCTAACGGACG	GCGCTCTGATGTACTGACCA
*Buffy*	ATACTGGGCTCCACCTCCAT	GCTCCCAAAAGCAAGCTCAC

### Radiochemistry, in vitro stability and in vivo biodistribution

#### Radiolabeling and radiochemical characterization

A lewis acid catalyst was used for the radioiodination of anle138b with ^131^I, as described by Racys et al. (2022) with minor modifications. Briefly, up to 350 MBq [^131^I]iodide in < 50 µl 0.01 M NaOH (IBSSO; GE Healthcare, Braunschweig, Germany) were added to a lead-shielded vial containing 150 µl anle138b (10 mM). After a reaction time of 2 h at room temperature in the dark, the product was purified using a PD-10 desalting column (Merck, Darmstadt, Germany). To assess the radiochemical purity (RCP) of [^131^I]I-anle138b, radio Thin Layer Chromatography (radio-TLC) measurements (miniGita; Elysia-Raytest, Straubenhardt, Germany) were performed. In short, 2 µl of the product were spotted on glass microfiber chromatography paper (stationary phase) impregnated with silicic acid (Agilent, Santa Clara, USA). A 2:1 mixture of Ethyl acetate/Hexane served as the mobile phase. In contrast to the unbound ^131^I, [^131^I]I-anle138b moves with the mobile phase. We consistently assessed RCPs of > 98%.

#### In vitro *stability*

Stability of [^131^I]I-anle138b was determined by incubating the radiolabeled compound in human serum at 37 °C. Aliquots thereof were analyzed at specified time points (0 h, 1 h, 4 h, 8 h and 24 h) by radio TLC regarding the present fraction of radioiodinated [^131^I]I-anle138b.

#### In vivo *biodistribution*

For the assessment of the in vivo biodistribution, 0–4 day old control flies were placed on food covered with 100 µl of radioiodinated [^131^I]I-anle138b (250 µM, 10 MBq) for 24 h as described above and subsequently subjected to 3 h of acute severe hypoxia (< 0.3 O_2_) or normoxia. We assessed the biodistribution of [^131^I]I-anle138b after 0 h and 24 h of reoxygenation. Therefore, the flies were snap-frozen and bodies and heads were separated for further analysis. After 3 washing steps with DMSO to remove possible residues of [^131^I]I-anle138b on the surface of the flies, the heads and bodies were separately lysed in Radioimmunoprecipitation assay (RIPA) buffer (#11873580001, Roche Basel, Switzerland) (10 µl per fly head). The samples were then transferred to gamma counter tubes and the quantitative [^131^I]I-anle138b biodistribution was measured using a gamma counter (Wizard^2^, PerkinElmer, Waltham, USA) with a protocol for ^131^I calibration. Activities were expressed as counts per minute. Each experiment was performed three times with 20 flies per condition and timepoint.

### Statistics

GraphPad Prism (version 10.3.1, San Diego, CA, USA) was used for data analysis and visualization. Each experiment consisted of three independent experiments, including three to four technical replicates (in total 180 to 240 flies per condition and genotype).

Normal distribution of residuals was assessed using the Shapiro–Wilk and D’Agostino-Pearson omnibus normality tests. Variance homogeneity was evaluated using the Bartlett test or Spearman’s rank correlation test for heteroscedasticity. Outliers were identified using the ROUT test. If normality or homogeneity tests showed significance, non-parametric tests were used instead of two-way ANOVA, as described in the corresponding legend. Data are presented as arithmetic means ± SEM, and statistical significance was set at *p* < 0.05. Asterisks indicate significance between groups, # indicates significance compared to the corresponding normoxia *p* < 0.05 (Fig. 5 c, d, f, g, i, j; Fig. 7 c, e-i). § indicates significance compared to the corresponding DMSO control *p* < 0.05 (Fig. 7 e-i). Detailed experimental data are provided in the legends.

## Results

### Acute hypoxic stimulation causes higher levels of α-Syn aggregation in comparison to repetitive and chronic hypoxia

**Fig. 1:**
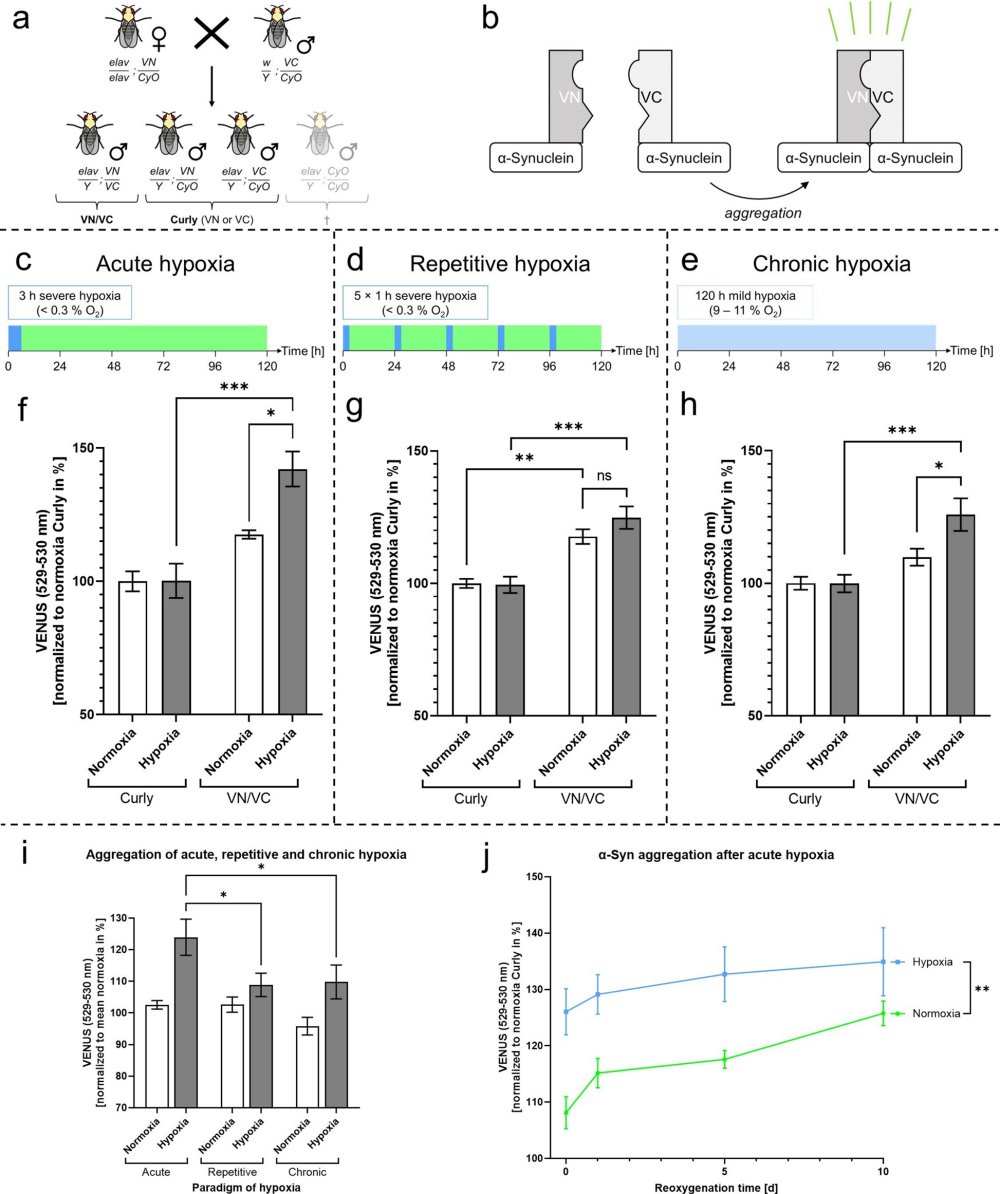
α-Syn aggregation in *Drosophila melanogaster* during reoxygenation period after acute, repetitive and chronic hypoxia. **a** Schematic illustration of the crossing strategy and genetic properties of the VN/VC and Curly *Drosophila melanogaster*. **b** Schematic illustration of the BiFC model α-Syn:VENUS. α-Syn molecules are marked with either the VN or the VC part of the VENUS protein. α-Syn dimerization leads to the completion of the fluorescent VENUS protein. The fluorescence levels represent α-Syn dimerization/aggregation. **c–e** Schematic workflow of acute (**c**), repetitive (**d**) and chronic (**e**) hypoxia/reoxygenation. **f** VENUS fluorescence levels representing α-Syn aggregation in VN/VC and Curly flies after 3 h of acute severe hypoxia (< 0.3% O_2_) or normoxia followed by a reoxygenation period of 120 h. **g** VENUS fluorescence levels representing α-Syn aggregation in VN/VC and Curly flies after repetitive severe hypoxia inductions (< 0.3% O_2_) over 5 days with alternating reoxygenation periods or normoxia. **h** VENUS fluorescence levels representing α-Syn aggregation in VN/VC and Curly flies after 120 h of chronic hypoxia (9–11% O_2_) or normoxia. **i** Comparison of the α-Syn aggregation levels of VN/VC flies after 120 h of reoxygenation following 3 h acute severe hypoxia (< 0.3% O_2_), repetitive severe hypoxia inductions (< 0.3% O_2_) over 5 days with alternating reoxygenation periods, 120 h of chronic mild hypoxia (9–11% O_2_) or normoxia. **j** VENUS fluorescence levels in VN/VC flies after 3 h of acute severe hypoxia or normoxia followed by a reoxygenation period of up to 10 days. Data are shown as means ± SEM of 3 independent experiments, including 3 technical replicates per genotype and experiment (total of 180 male flies for each genotype and condition). Multiple comparisons two-way ANOVA followed by Tukey post-hoc (**f**–**i**), paired t-test (**j**). *p* < 0.05. *p < 0.05, **p < 0.01, ***p < 0.001

To investigate whether hypoxia leads to neuronal α-Syn aggregation and to examine the impact of different hypoxic stimulation paradigms on α-Syn aggregation levels, we utilized the BiFC system α-Syn:VENUS. Fluorescence occurs when aggregation, at least dimerization, of α-Syn tagged with both VN and VC fragments completes the VENUS protein (Fig. 1b). We performed a Thioflavin T assay to validate that the VENUS signal correlates with the formation of α-Syn fibrils/aggregates (Pearson *r* = 0.98, *p* < 0.001, Fig. 9a–c). We subjected *Drosophila melanogaster* with pan-neuronal expression of either both BiFC fragments (VN/VC) or only one BiFC fragment (Curly) to acute, repetitive, or chronic hypoxia and compared the VENUS fluorescence levels (529–530 nm wavelength), representing α-Syn aggregation after 120 h (5 days) of reoxygenation (Fig. 1a, c–e).

α-Syn aggregation levels were significantly higher after acute and chronic hypoxia compared to the corresponding normoxia following 120 h of reoxygenation in VN/VC flies (*p* < 0.0385, Fig. 1f–h). Acute hypoxia led to significantly higher post-hypoxic aggregation levels in comparison to the repetitive and chronic hypoxia paradigm (*p* < 0.0405, Fig. 1i). To investigate whether α-Syn aggregation occurs primarily during hypoxia or during the reoxygenation phase, we measured VENUS fluorescence levels immediately after acute severe hypoxia (< 0.3% O_2_) and over a reoxygenation period of 10 days. The α-Syn aggregation levels were significantly elevated immediately after hypoxia and persisted markedly elevated throughout the reoxygenation period of 10 d in comparison to the corresponding normoxic control (*p* = 0.0046, Fig. 1j).

In summary, acute and chronic hypoxia caused a significant elevation of α-Syn aggregation with acute severe hypoxia resulting in the highest α-Syn aggregation levels.

### Acute severe hypoxia (< 0.1% O_2_) increases aggregation levels after 24 h of reoxygenation in HEK-293 cells

**Fig. 2:**
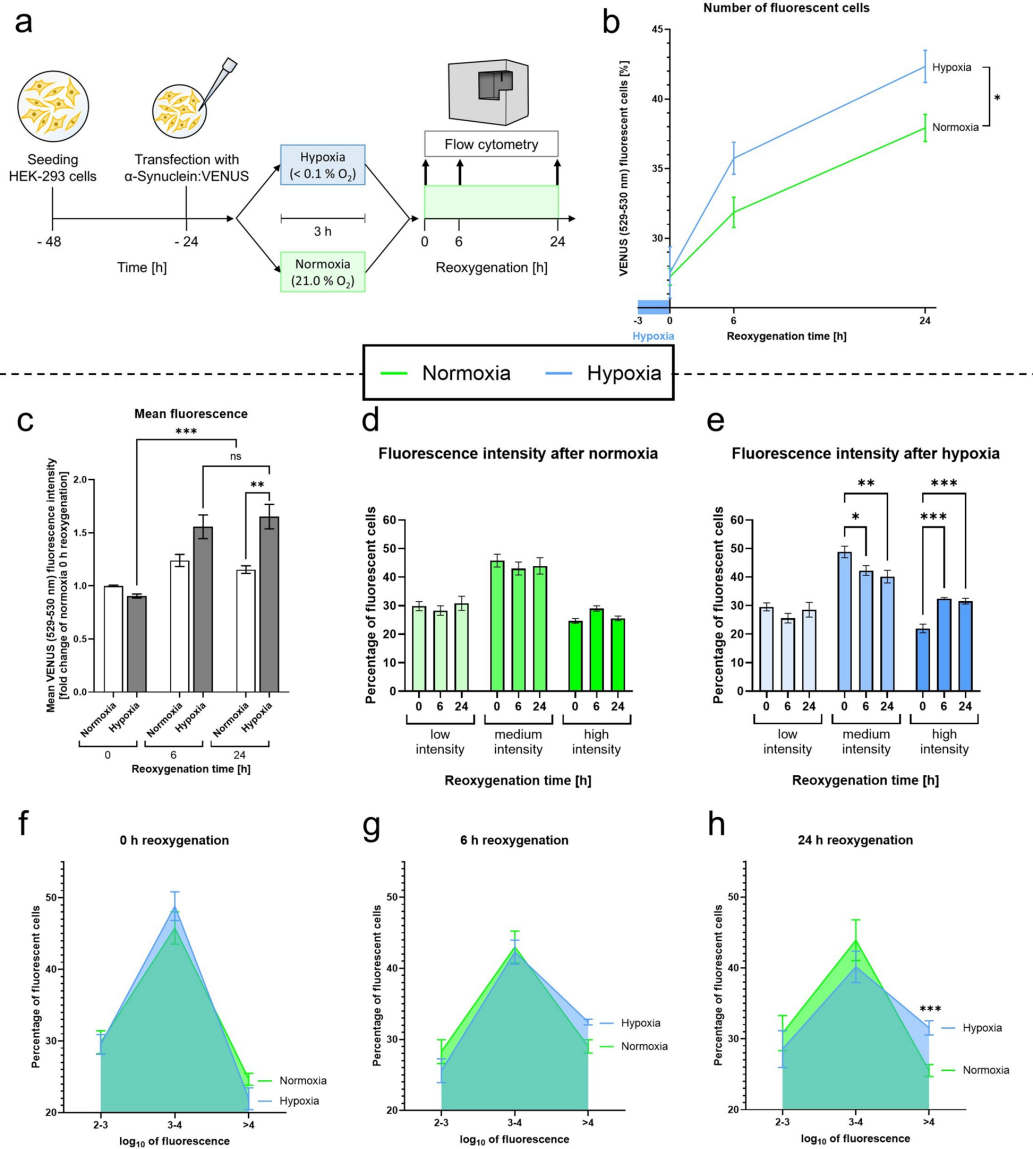
α-Syn aggregation in HEK-293 cells after acute severe hypoxia and during reoxygenation. **a** Schematic illustration of the experimental protocol. **b** Post-hypoxic percentage of fluorescent HEK-293 cells in relation to the total cell count under normoxic and hypoxic conditions during a reoxygenation period of up to 24 h. **c** Mean fluorescence intensity of HEK-293 cells under normoxic and hypoxic conditions during a reoxygenation period of up to 24 h. **d–e** Time-dependent intensity distribution of VENUS-positive HEK-293 cells after 3 h of normoxia (**d**) or acute severe hypoxia (**e**) over a reoxygenation period of 24 h. **f–h** Percentage distribution of the fluorescence intensity of the VENUS-positive HEK-293 cells after 3 h of hypoxia followed by a reoxygenation period of 0 h (**f**), 6 h (**g**) and 24 h (**h**). Data are shown as means ± SEM of 3 independent experiments, including 3 technical replicates per condition and experiment. Šídák's multiple comparisons two-way ANOVA (**b**), multiple comparisons two-way ANOVA followed by Tukey post-hoc (**c**–**e**), multiple t-test (**f**–**h**). *p < 0.05, **p < 0.01, ***p < 0.001

To analyze the impact of acute severe hypoxia (< 0.1% O_2_) on α-Syn aggregation on a single cell level, we subjected VN/VC-transfected HEK-293 cells to 3 h of acute hypoxia and quantified the post-hypoxic VENUS-fluorescence levels per cell using a flow cytometry analysis (Figs. 2a, 8a–c).

Similar to *Drosophila melanogaster*, the HEK-293 cells displayed a time-dependent increase in the number of cells with detectable α-Syn aggregates under both normoxic and hypoxic conditions. Notably, we observed a progressively growing divergence between the hypoxia and normoxia groups, starting with comparable α-Syn aggregation levels immediately after the hypoxic period and culminating in a significantly higher proportion of fluorescent cells in the hypoxia group after 24 h of reoxygenation compared to the normoxia control (*p* = 0.0035, Fig. 2b).

While the mean α-Syn aggregation levels of the normoxia-treated cells were only mildly elevated throughout the reoxygenation period, post-hypoxic reoxygenation markedly increased α-Syn aggregation levels (*p* = 0.0003 at 6 h, *p* < 0.0001 at 24 h of reoxygenation). After 24 h of reoxygenation, we observed significantly higher mean α-Syn aggregation levels in cells subjected to hypoxia compared to the normoxia group (*p* = 0.0035, Fig. 2c). Thioflavin S staining confirmed that acute severe hypoxia causes a significant increase of fibrillar α-Syn structures throughout a reoxygenation period of 24 h (*p* < 0.0366, Fig. 9d–f).

To distinguish between cells exhibiting varying levels of α-Syn aggregation, we compared cell populations with low, medium and high fluorescence intensity throughout the reoxygenation period following hypoxia or normoxia.

While the proportion of these populations remained constant over the course of reoxygenation in the normoxia group, the hypoxia group exhibited a significant decline in the percentage of cells exhibiting medium fluorescence intensity over time with a concomitant increase in the percentage of cells exhibiting high intensity, suggesting a shift from the medium- to the high-intensity group (*p* < 0.0243, Fig. 2d–e).

After 24 h of reoxygenation, the proportion of cells with high levels of α-Syn aggregation was significantly higher in the post-hypoxic group compared to the normoxia group (*p* = 0.0003, Fig. 2f–h).

In conclusion, acute hypoxia led to a gradual elevation of α-Syn aggregation levels in HEK-293 cells during the reoxygenation period compared to the corresponding normoxia control. This increase in α-Syn aggregation levels was in particular driven by cells displaying medium to high levels of α-Syn aggregation.

### Post-hypoxic α-Syn aggregation increases mortality rates and shortens the overall longevity of *Drosophila melanogaster*

To analyze the impact of α-Syn on the survival of *Drosophila melanogaster*, we subjected flies expressing α-Syn (VN/VC flies) and elav-Gal4 flies without α-Syn expression (control flies) to different durations of hypoxia and assessed mortality rates and longevity.

**Fig. 3:**
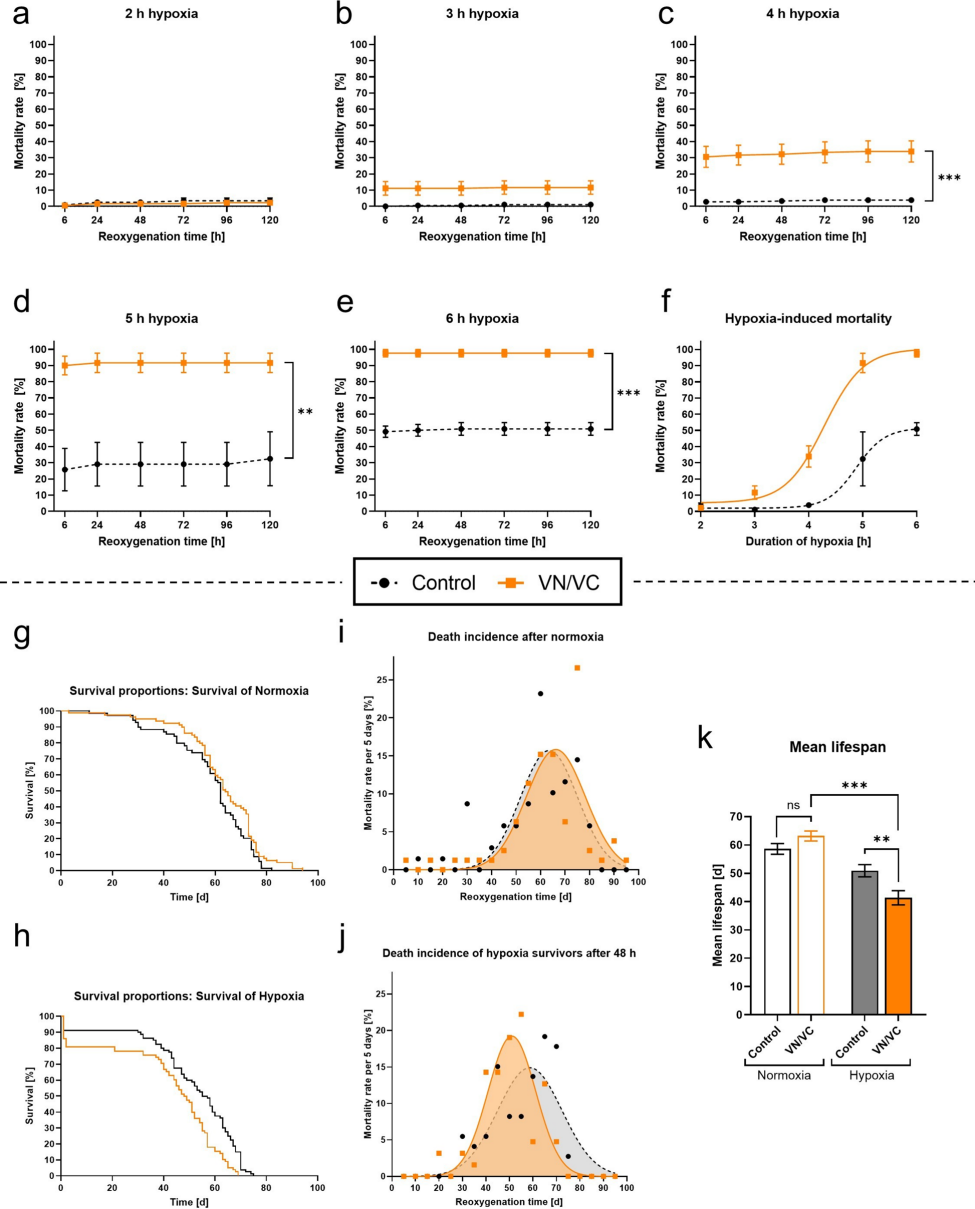
Mortality and longevity of VN/VC and control flies after acute severe hypoxia. **a–e** Death rates of VN/VC and control flies after 2–6 h of severe hypoxia (< 0.3% O_2_) followed by a reoxygenation period of 120 h. **f** Nonlinear regression of the hypoxia duration-dependent death rates of VN/VC and control flies after 120 h of reoxygenation. **g–h** Kaplan–Mayer-curves of VN/VC and control flies subjected to 3 h of normoxia (**g**) or hypoxia (**h**). **i–j** Death incidence per 5 days of VN/VC and control flies surviving the first 48 h after acute hypoxia or normoxia. **k** Mean lifespan of VN/VC and control flies after acute severe hypoxia or normoxia. Data are shown as means ± SEM of 3 independent experiments, including 3 technical replicates per genotype and experiment (total of 180 male flies for each genotype and condition) (**a**–**f**), 4 independent experiments with 20 male flies per genotype and condition (total of 80 male flies for each genotype and condition) (**g**–**k**). Šídák's multiple comparisons two-way ANOVA (**a**–**e**), multiple comparisons two-way ANOVA followed by Tukey post-hoc (**k**). *p < 0.05, **p < 0.01, ***p < 0.001

Increasing duration of hypoxia gradually elevated mortality rates in both VN/VC and control flies. However, VN/VC flies presented significantly higher mortality rates after 4 to 6 h of hypoxia during a reoxygenation period of 120 h compared to control flies (*p* < 0.0041, Fig. 3a–e). We observed that all hypoxia-induced acute deaths occurred within the first 24 h of reoxygenation. A nonlinear regression analysis of the mortality rates after 120 h of post-hypoxic reoxygenation revealed overall higher death rates in VN/VC flies compared to control flies (Fig. 3f).

Under normoxic conditions we observed no significant difference in lifespan between VN/VC (median survival: 64 d) and control (median survival: 62 d) *Drosophila melanogaster*. 3 h of acute hypoxia reduced mean life expectancy in both VN/VC and control flies compared to normoxia (*p* < 0.0001 in VN/VC). Notably, VN/VC flies exhibited a significantly shorter post-hypoxic mean longevity (median survival: 48.5 d) than control flies (median survival: 56 d) (*p* = 0.0068, Fig. 3g, h, k).

To exclude the impact of the acute post-hypoxic mortality (first 48 h of reoxygenation) on the overall lifespan, we monitored the death incidences starting 48 h after hypoxia (Fig. 3i, j). Under normoxic conditions, we observed no differences in the time-dependent death incidence between VN/VC and control flies. However, upon hypoxia, VN/VC flies presented a higher maximum mortality rate at an earlier timepoint, indicating that VN/VC flies died in a shorter and earlier timeframe than control flies (Fig. 3i, j).

In conclusion, acute severe hypoxia increased post-hypoxic mortality rates and declined life expectancy in both VN/VC and control flies. However, α-Syn aggregation coincided with higher mortality rates and lower longevity after hypoxia compared to control flies.

### Post-hypoxic α-Syn aggregation reduces the activity of *Drosophila melanogaster* and prolongs the post-hypoxic recovery time

**Fig. 4:**
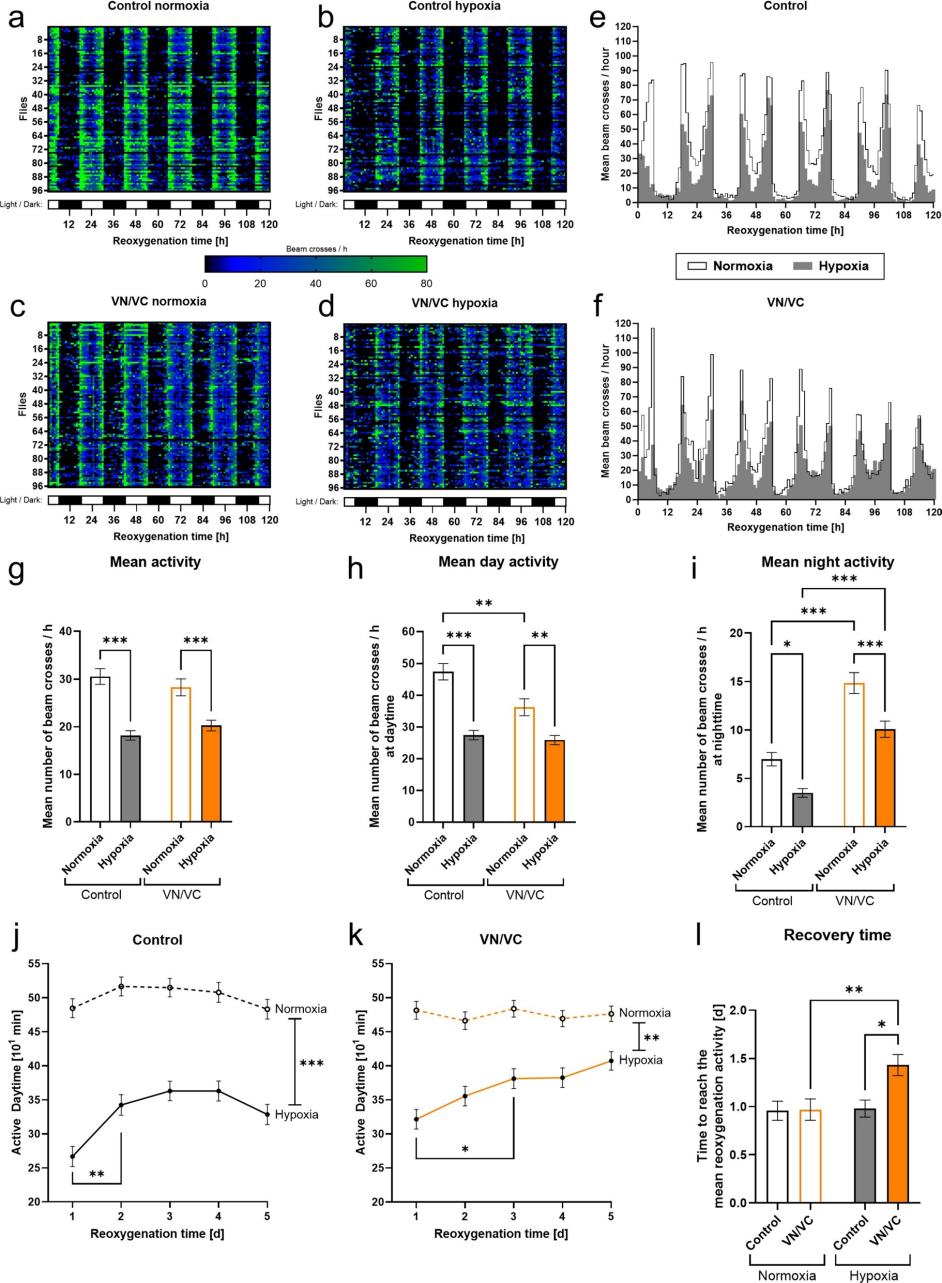
Activity, sleep cycle and recovery of VN/VC and control flies after acute severe hypoxia. **a–d** Heatmaps displaying the activity of VN/VC and control flies after 3 h of severe hypoxia (< 0.3% O_2_) or normoxia (21% O_2_) followed by 120 h of reoxygenation. Each cell represents the absolute value of beam crosses per hour per fly, represented by color. Black: no activity, blue: moderate activity, green: high activity. **e–f** Actograms displaying the mean number of beam crosses per hour of VN/VC and control flies after 3 h of severe hypoxia (< 0.3% O_2_) or normoxia (21% O_2_) followed by 120 h of reoxygenation. **g** Mean activity levels of control and VN/VC flies during 120 h of reoxygenation following 3 h of severe hypoxia (< 0.3% O_2_) or normoxia. **h** Mean day activity of control and VN/VC flies during 120 h of reoxygenation following 3 h of severe hypoxia (< 0.3% O_2_) or normoxia. **i** Mean night activity of control and VN/VC flies during 120 h of reoxygenation following 3 h of severe hypoxia (< 0.3% O_2_) or normoxia. **j–k** Average amount of active minutes per day of control (**j**) and VN/VC (**k**) flies after 3 h of hypoxia or normoxia. **l** Recovery time as the time needed to reach the mean reoxygenation activity after 3 h of hypoxia or normoxia. Data are shown as means ± SEM of 3 independent experiments (total of 96 male flies for each genotype and condition). Multiple comparisons two-way ANOVA followed by Tukey post-hoc (**g**–**i**, **l**), Šídák's multiple comparisons two-way ANOVA for comparison of normoxia and hypoxia (**j**–**k**), multiple comparisons two-way ANOVA followed by Tukey post-hoc for comparison of timepoints (**j**–**k**). *p < 0.05, **p < 0.01, ***p < 0.001

As synucleinopathies are attributed with both cognitive and locomotor impairments, we monitored the impact of hypoxia on spontaneous activity, day-night-cycle and post-hypoxic activity recovery time of *Drosophila melanogaster*. Therefore, we subjected VN/VC and control flies to 3 h of hypoxia or normoxia and assessed their activity levels in an automated Drosophila Activity Monitor (DAM) system for 5 days on a 12 h light/dark cycle (Fig. 4a–f).

Under normoxic conditions we observed no differences in the mean activity levels between VN/VC and control flies (Fig. 4g). However, VN/VC flies exhibited significantly lower daytime activity levels and significantly higher nighttime activity levels compared to control flies even under normoxic conditions (*p* < 0.001, Fig. 4h–i).

Hypoxia led to a significant decrease of the mean activity as well as day and night activity levels of both VN/VC and control flies compared to normoxia control (*p* < 0.001, Fig. 4g–i). In VN/VC flies, the impact of hypoxia appeared to be more pronounced on the nighttime activity, while control flies displayed a greater impairment in post-hypoxic daytime activity (Fig. 4h–i).

After normoxia we observed constant activity durations in both VN/VC and control flies over the entire 5-day reoxygenation period. Hypoxia significantly decreased the activity duration in both genotypes, exhibiting the lowest activity levels on the first post-hypoxic day, followed by a restoration period during which the daily activity duration increased again (*p* < 0.0012, Fig. 4j–l). However, in VN/VC flies, a significant increase of activity levels was observed on day 3, a day later than in control flies, demonstrating an overall prolonged recovery time compared to control flies (*p* < 0.0143, Fig. 4j–l).

In conclusion, hypoxia decreased the activity levels of both control and VN/VC flies. Hypoxia-induced α-Syn aggregation corresponded with changes in the day/night-activity levels and prolonged the recovery time in *Drosophila melanogaster*.

### Post-hypoxic α-Syn aggregation markedly activates the PERK branch of the UPR

**Fig. 5:**
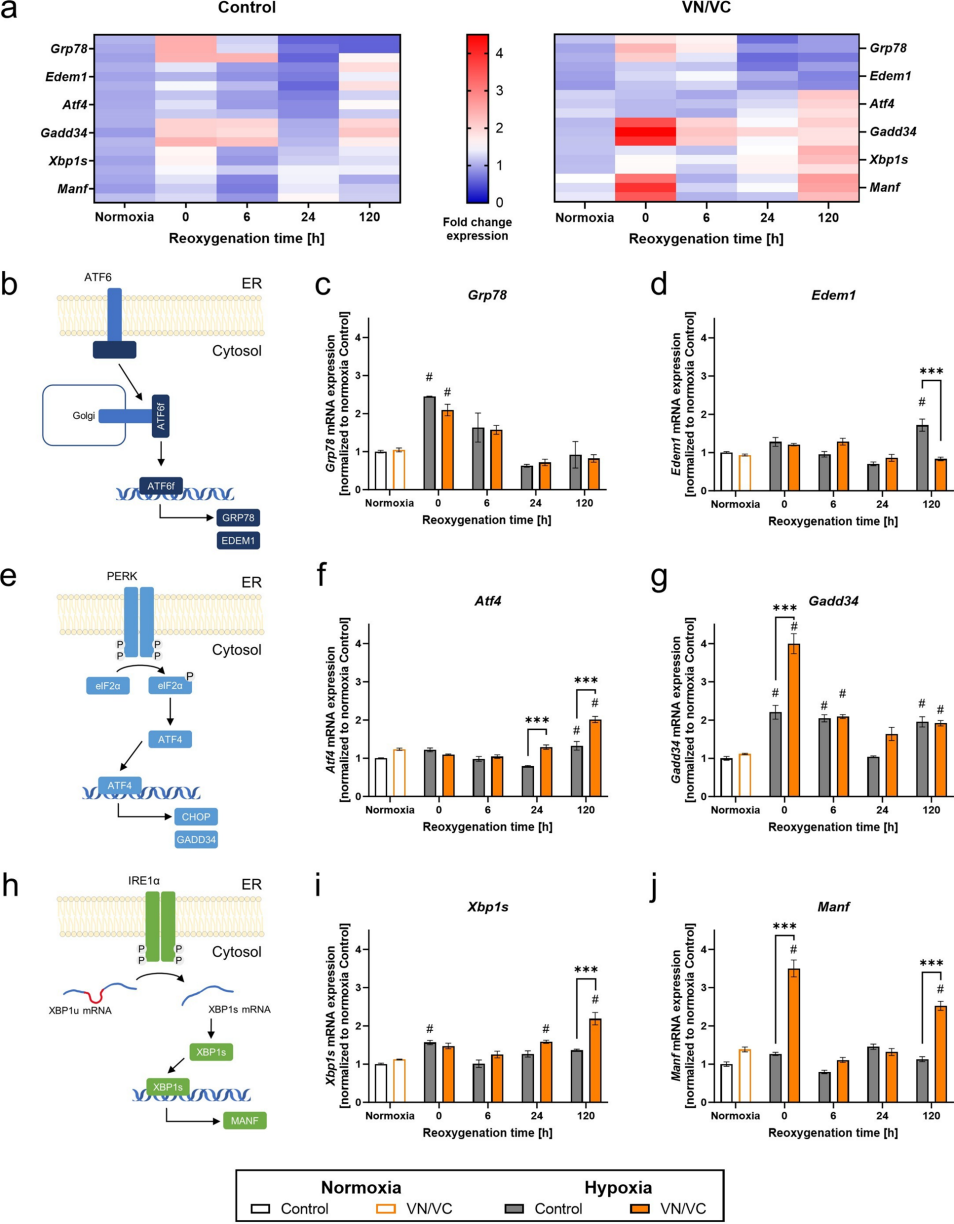
Post-hypoxic mRNA levels of the UPR pathway in VN/VC and control flies during reoxygenation. **a** Heatmap displaying the mRNA levels of *Grp78*, *Edem1*, *Atf4*, *Gadd34*, *Xbp1s* and *Manf* in VN/VC and control flies at different post-hypoxic reoxygenation timepoints (0, 6, 24 and 120 h after hypoxia) as a summary of the results of the graphs **c**, **d**, **f**, **g**, **i**, **j**. Target genes are presented as ratios to the constitutive gene *eEF1α2* and normalized to the control normoxia. **b, e, h** Schematic illustration of the three branches of the UPR (ATF6, PERK, IRE1α) displaying the investigated markers. **c, d, f, g, i, j** Graphs showing the mRNA expression levels of markers of the ATF6 (**c**–**d**), PERK (**f**–**g**) and IRE1α (**i**–**j**) branch of the UPR**.** Target genes are presented as ratios to the constitutive gene *eEF1α2* and normalized to the control normoxia. Data are shown as means ± SEM of 3 independent experiments, including 3 technical replicates per genotype and experiment (total of 180 male flies for each genotype and condition). Multiple comparisons two-way ANOVA followed by Tukey post-hoc. # indicates significance compared to the corresponding normoxia *p* < 0.05. *p < 0.05, **p < 0.01, ***p < 0.001

To analyze the influence of α-Syn on the post-hypoxic activation of the three UPR branches, we compared the expression levels of *Grp78, Edem1* (ATF6 arm)*, Atf4, Gadd34* (PERK arm)*, Xbp1s* and *Manf* (IRE1α arm) in VN/VC and control flies (Fig. 5a, b, e, h).

Hypoxia induced an upregulation of all three UPR branches over the observation period of 120 h, peaking directly after hypoxia at 0 h of reoxygenation and returning to normoxic levels after 24 h of reoxygenation in both genotypes. While the control flies displayed higher expression levels of the ATF6 related genes *Grp78* and *Edem1* during reoxygenation, α-Syn aggregation corresponded with a stronger post-hypoxic upregulation of the PERK markers *Gadd34* and *Atf4* as well as the IRE1α markers *Xbp1s* and *Manf* (*p* < 0.0195). Notably, while control flies only displayed a mild upregulation (one- to twofold), VN/VC flies presented a strong upregulation (about fourfold) of the abovementioned UPR markers with significantly higher expression levels of *Gadd34* and *Atf4* (PERK branch) as well as *Manf* (IRE1α branch) after hypoxia compared to control flies (*p* < 0.0001, Fig. 5e–j).

In summary, hypoxia activated all three branches of the UPR. However, there was a stronger upregulation of the PERK and IRE1α branches in flies with α-Syn, while control flies primarily exhibited an upregulation of the ATF 6 branch.

### Post-hypoxic elevated levels of aggregation inhibitor anle138b improve longevity and cognition of *Drosophila melanogaster*

**Fig. 6:**
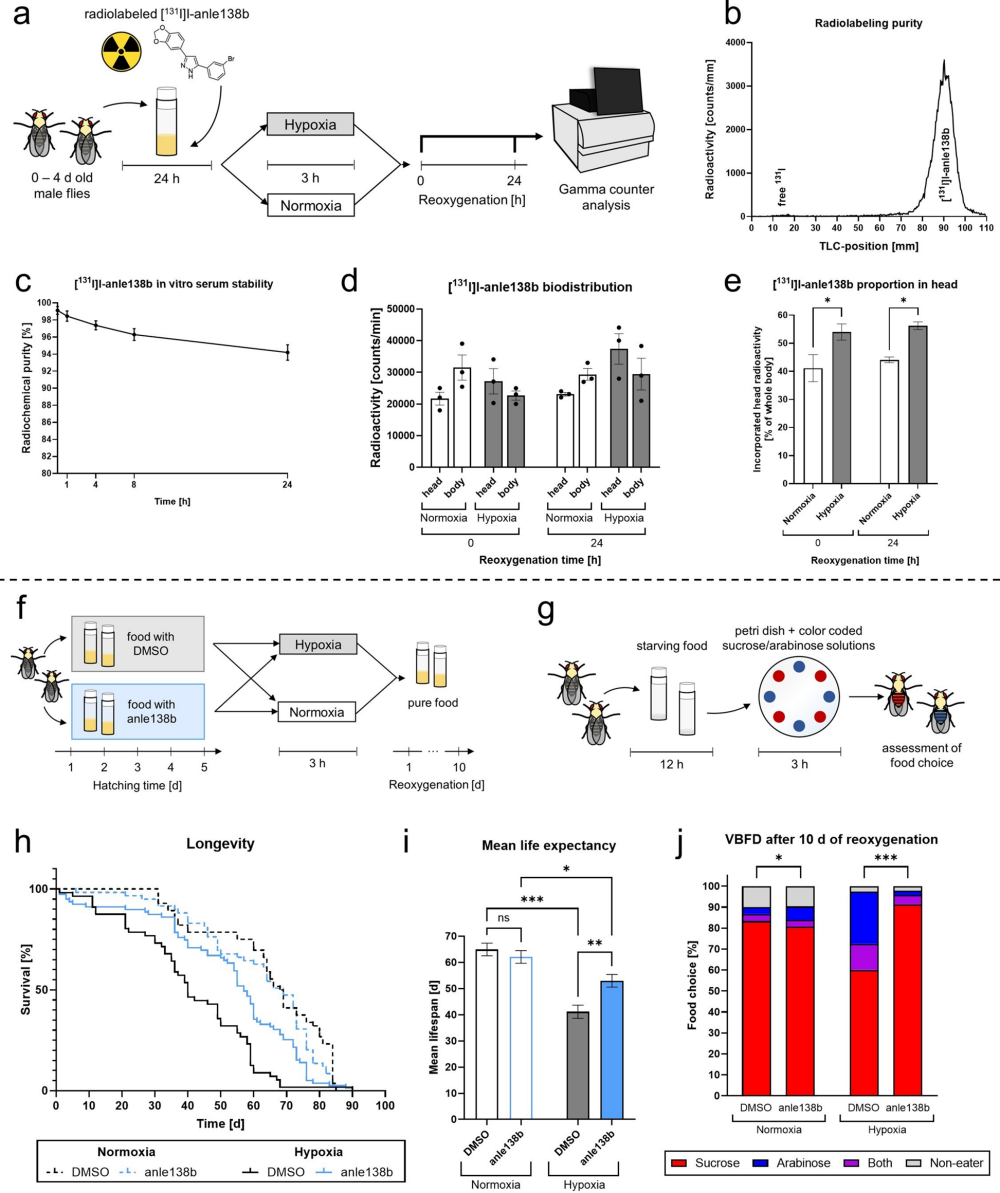
Radiolabeling and biodistribution of [^131^I]I-anle138b and its impact on post-hypoxic decision-making and longevity. **a** Schematic illustration of the administration protocol with [^131^I]I-anle138b and analysis. **b** Radiolabeling purity of [^131^I]I-anle138b, represented by a radio-TLC chromatogram. **c** [^131^I]I-anle138b in vitro serum stability over an observation period of 24 h. **d** [^131^I]I-anle138b biodistribution in head and body of control flies after 0 h and 24 h of reoxygenation following 3 h of hypoxia or normoxia. **e** Proportion of [^131^I]I-anle138b in the head of control flies after 0 h and 24 h of reoxygenation following 3 h of hypoxia or normoxia. **f** Schematic illustration of the treatment protocol with DMSO and anle138b. **g** Schematic illustration of the Value Based Feeding Decision (VBFD) assay. **h** Lifespan of DMSO- or anle138b-treated VN/VC flies after exposure to 3 h of acute hypoxia or normoxia. **i** Mean life expectancy of VN/VC flies treated with DMSO or anle138b after 3 h of acute hypoxia or normoxia. **j** Value-based feeding decision (VBFD) of DMSO- or anle138b-treated VN/VC flies after 3 h of acute severe hypoxia (< 0.3% O_2_) or normoxia after 10 d of reoxygenation. Data are shown as means ± SEM of 3 independent experiments (total of 60 male flies for each timepoint and condition). Šídák's multiple comparisons two-way ANOVA (**e**), multiple comparisons two-way ANOVA followed by Tukey post-hoc (**i**), Fisher’s exact test for Sucrose vs. no Sucrose (**j**). *p < 0.05, **p < 0.01, ***p < 0.001

To assess the impact of hypoxia on the biodistribution of anle138b in *Drosophila melanogaster* we placed flies on food supplemented with radiolabeled [^131^I]I-anle138b 24 h prior to hypoxia or normoxia and assessed the radioactivity in heads and bodies during a reoxygenation period of 24 h in a Gamma counter (Fig. 6a).

We achieved a radiolabeling purity of [^131^I]I-anle138b of over 98% in every batch (Fig. 6b). Moreover, the serum stability of our [^131^I]I-anle138b in human serum was approximately 94% after 24 h (Fig. 6c).

We observed similar radioactivity levels in the heads and bodies of the flies following both hypoxia and normoxia over a reoxygenation period of 24 h (Fig. 6d). Interestingly, hypoxia significantly increased the fraction of [^131^I]I-anle138b accumulated in the head compared to the normoxia control at both 0 h and 24 h of reoxygenation (*p* < 0.0372, Fig. 6e).

To investigate the impact of the aggregation inhibitor anle138b on the flies’ mortality, longevity and decision-making ability, we placed VN/VC flies on food supplemented with anle138b or DMSO and subjected them to acute severe hypoxia or normoxia (Fig. 6f).

Treatment with anle138b exhibited no effect on the mortality rate of VN/VC flies during the first 5 days of reoxygenation following 2 to 6 h of hypoxia (Fig. 10a–f).

Under normoxic conditions, treatment with anle138b did not significantly affect the lifespan of the flies (median lifespan after DMSO treatment: 68.5 d, median lifespan after anle138b treatment: 69 d). Hypoxia caused a significant decline in the mean life expectancy of both the DMSO and the anle138b treated flies compared to normoxia (*p* < 0.04). However, anle138b treatment significantly improved the life expectancy after hypoxia compared to the DMSO vehicle control (*p* = 0.003). While flies subjected to DMSO exhibited a median lifespan of 40 d, anle138b treated flies lived for a median of 57 d, suggesting that hypoxia-induced α-Syn aggregation causes a significant reduction in life expectancy (Fig. 6h–i).

To analyze the effect of anle138b treatment on the post-hypoxic cognitive function of VN/VC flies, we analyzed the flies’ decision-making ability in a Value Based Feeding Decision (VBFD) assay after 10 d of reoxygenation. Here, we assessed the flies’ food choice between sucrose, which is well-metabolizable by flies, and arabinose, which flies cannot metabolize well (Fig. 6g). Under normoxic conditions, we observed no difference in the food choice between the DMSO and anle138b-treated flies. Hypoxia caused a significant decline in the ability to choose sucrose compared to normoxia control in the DMSO vehicle group (*p* = 0.0398). However, when treated with anle138b, the flies displayed a significantly improved post-hypoxic decision-making ability compared to the DMSO group, choosing the sucrose food as often as the normoxia control 10 d after the hypoxia (*p* = 0.0008, Fig. 6j).

In conclusion, hypoxia significantly increased the uptake of [^131^I]I-anle138b in the head of the flies. Anle138b did not affect the acute mortality rate after hypoxia, but markedly improved post-hypoxic longevity and decision-making of *Drosophila melanogaster*.

### Anle138b decreases post-hypoxic α-Syn aggregation and attenuates the post-hypoxic upregulation of the PERK branch of the UPR

Since the PERK branch of the UPR was highly upregulated upon hypoxia, we sought to investigate how hypoxia-induced α-Syn aggregation and PERK activation influence each other. Therefore, we assessed the impact of aggregation inhibitor anle138b and PERK inhibitor GSK2606414 (GSK) on α-Syn aggregation levels and PERK activation.

**Fig. 7:**
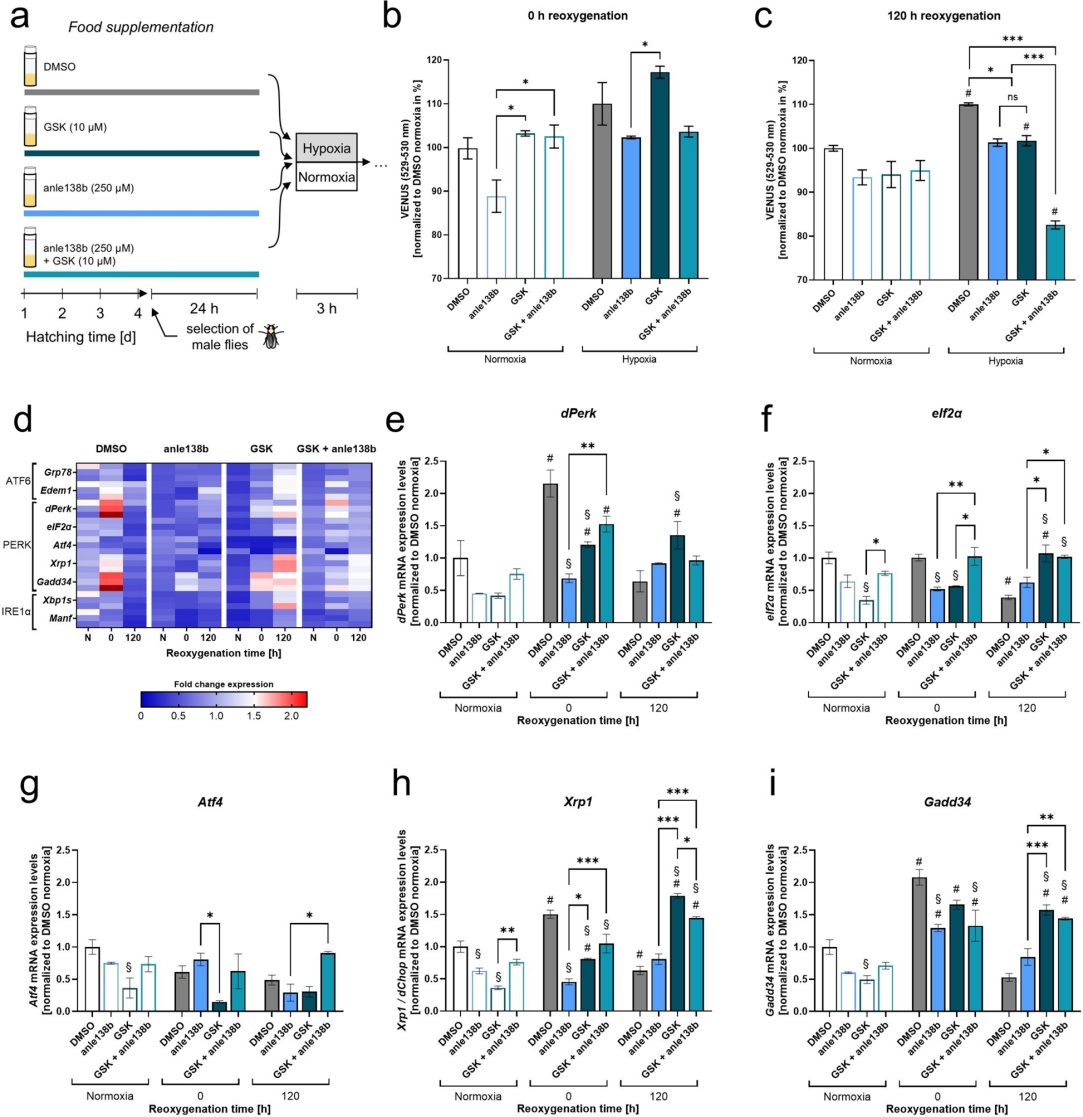
Post-hypoxic α-Syn aggregation levels and UPR mRNA levels after anle138b or GSK2606414 treatment. **a** Schematic illustration of the treatment protocol with DMSO, anle138b, GSK2606414 or GSK2606414 + anle138b. **b–c** Post-hypoxic and post-normoxic VENUS fluorescence levels representing α-Syn aggregation after treatment with DMSO, anle138b, GSK or GSK + anle138b according to the treatment protocol in **a** after a reoxygenation period of 0 h (**b**) and 120 h (**c**). **d** Heatmap displaying the post-hypoxic mRNA levels of *Grp78*, *Edem1*, *dPerk*, *eIF2α*, *Atf4*, *Xrp1*, *Gadd34*, *Xbp1s* and *Manf* in VN/VC flies at different reoxygenation timepoints (0 and 120 h after hypoxia) after treatment with DMSO, anle138b, GSK or GSK + anle138b prior to the hypoxia. Target genes are presented as ratios to the constitutive gene *Actin5c* and normalized to the control normoxia. **e–i** Graphs showing the post-hypoxic mRNA expression levels of markers of the PERK branch of the UPR (*dPerk*, *eIF2α*, *Atf4*, *Xrp1*, *Gadd34*) in VN/VC flies after treatment with DMSO, anle138b, GSK or GSK + anle138b. Target genes are presented as ratios to the constitutive gene *Actin5c* and normalized to the control normoxia. Data are shown as means ± SEM of 3 independent experiments, including 3 technical replicates per genotype and experiment (total of 180 male flies for each treatment, timepoint and condition). Multiple comparisons two-way ANOVA followed by Tukey post-hoc (**b**–**i**). # indicates significance compared to the corresponding normoxia *p* < 0.05. § indicates significance compared to the corresponding DMSO control *p* < 0.05. *p < 0.05, **p < 0.01, ***p < 0.001

**Fig. 8:**
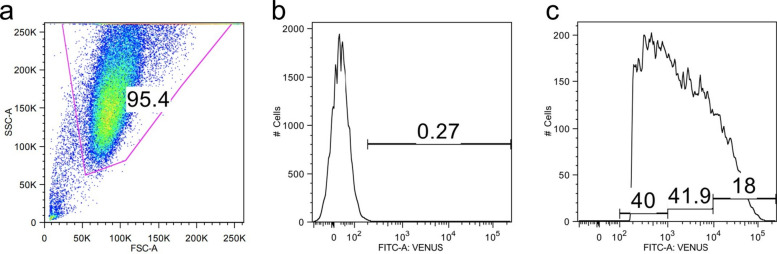
Gating strategy for flow cytometry analysis of HEK-293 cells. **a**–**c** Gating strategy for flow cytometry analysis of α-Syn:VENUS transfected HEK-293 cells. Live cells were gated based on forward and side scatter (FSC/SSC) characteristics to exclude debris and dead cells (**a**). An empty plasmid control was used to exclude background (**b**), and the signal from experimental samples was divided into low (10^2^–10^3^), medium (10^3^–10^4^) and high (> 10^4^) intensity fluorescence for further analysis (**c**)

**Fig. 9:**
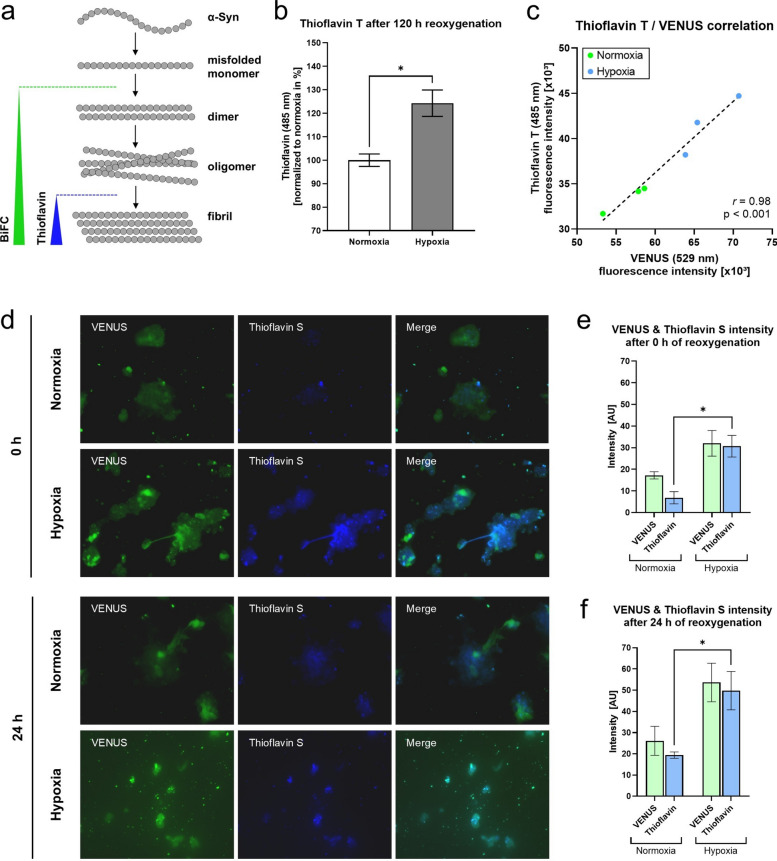
Post-hypoxic Thioflavin and VENUS fluorescence in *Drosophila melanogaster* and HEK-293 cells. **a** Schematic illustration of structures detected by BiFC and Thioflavin. While BiFC is very sensitive to early oligomerization, Thioflavin specifically marks fibrillic structures. **b** Thioflavin T fluorescence levels representing fibrillic α-Syn aggregates in VN/VC flies after 3 h of acute severe hypoxia (< 0.3% O_2_) or normoxia followed by a reoxygenation period of 120 h. **c** Correlation between Thioflavin T and VENUS fluorescence signal in *Drosophila melanogaster*. Pearson *r*. **d** Representative fluorescence imaging of VENUS (green) and Thioflavin S (blue) staining in HEK-293 cells after 3 h of acute severe hypoxia or normoxia followed by a reoxygenation period of 24 h. **e–f** Mean VENUS & Thioflavin S fluorescence intensity after 0 h (**e**) and 24 h (**f**) of reoxygenation following 3 h of acute severe hypoxia or normoxia in HEK-293 cells. Data are shown as means ± SEM of 3 independent experiments. Unpaired t-test (**b**). Pearson *r* (**c**). Multiple comparisons two-way ANOVA followed by Tukey post-hoc (**e**, **f**). *p < 0.05

**Fig. 10:**
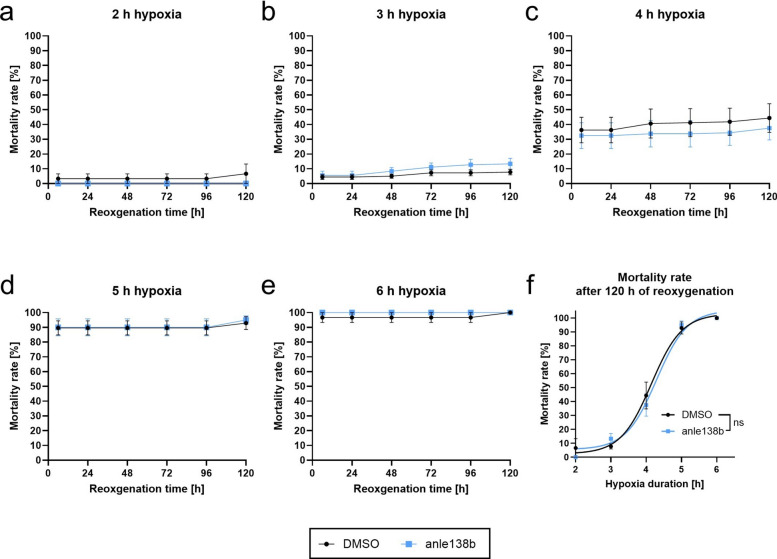
Post-hypoxic mortality of VN/VC flies after anle138b or DMSO treatment during 120 h of reoxygenation. **a-e** Mortality rates of VN/VC flies treated with anle138b or DMSO vehicle during a reoxygenation period of 120 h following 2–6 h of acute severe hypoxia (< 0.3% O_2_).** f** Nonlinear regression of the hypoxia duration-dependent death rates of VN/VC flies after treatment with DMSO or anle138b after 120 h of reoxygenation. Data are shown as means ± SEM of 3 independent experiments, including 3 technical replicates per experiment (total of 180 male flies for each treatment and condition). Unpaired multiple t-test (**a**–**f**). ns indicates no significance

VN/VC flies were fed food supplemented with DMSO or anle138b for 4 days. Out of those, male flies were separated and placed on food supplemented with DMSO, anle138b, GSK or the combination of anle138b and GSK for another 24 h followed by 3 h of acute severe hypoxia (Fig. 7a). We measured α-Syn aggregation after 0 h and 120 h of reoxygenation.

**Fig. 11:**
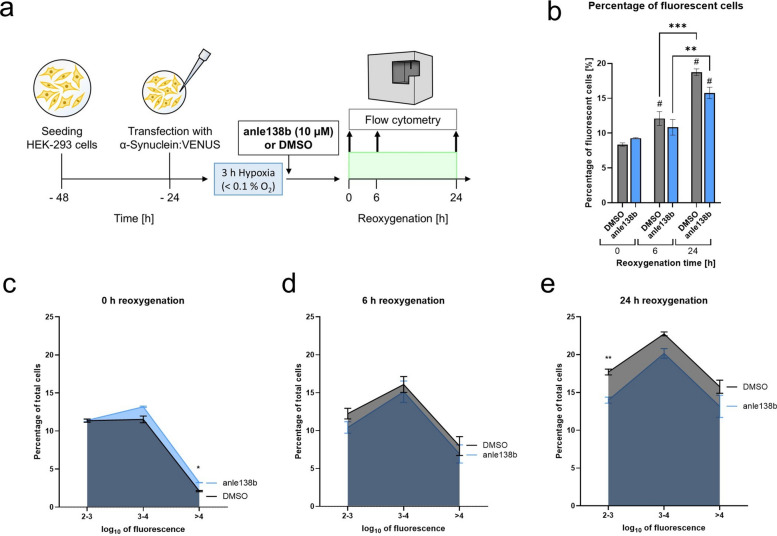
Hypoxia-induced α-Syn aggregation in HEK-293 cells after post-hypoxic treatment with anle138b or DMSO.** a** Schematic illustration of the experimental protocol. **b** Percentage of fluorescent HEK-293 cells, representing the number of cells exhibiting aggregation**. c–e** Percentage of HEK-293 cells with low, medium and high fluorescence intensity after post-hypoxic anle138b or DMSO treatment during a reoxygenation period of 0 h (**c**), 6 h (**d**) and 24 h (**e**). Data are shown as means ± SEM of 3 independent experiments, including 3 technical replicates per conditions and experiments. multiple t-test. # indicates significance compared to corresponding value at 0 h of reoxygenation *p* < 0.05. **p < 0.01, ***p < 0.001

Hypoxia caused an increase of α-Syn aggregation levels in the DMSO group during early and late reoxygenation time points, resulting in significantly higher α-Syn aggregation levels after 120 h of reoxygenation compared to the corresponding normoxia (*p* = 0.007, Fig. 7b–c). Treatment with the aggregation inhibitor anle138b reduced post-hypoxic α-Syn aggregation levels throughout the reoxygenation period, exhibiting significantly lower aggregation levels after 120 h of reoxygenation compared to the DMSO group (*p* = 0.0444). Following hypoxia, GSK-treated flies initially exhibited a small transient increase in α-Syn aggregation, which significantly decreased after 120 h of reoxygenation compared to the DMSO group (*p* = 0.0299, Fig. 7b–c). The combination of both inhibitors further mitigated α-Syn aggregation after 120 h of reoxygenation (*p* < 0.0001, Fig. 7c). Interestingly, treatment with anle138b after hypoxia was able to reduce early accumulation of α-Syn aggregates during a reoxygenation period of 24 h in HEK-293 cells (Fig. 11).

**Fig. 12:**
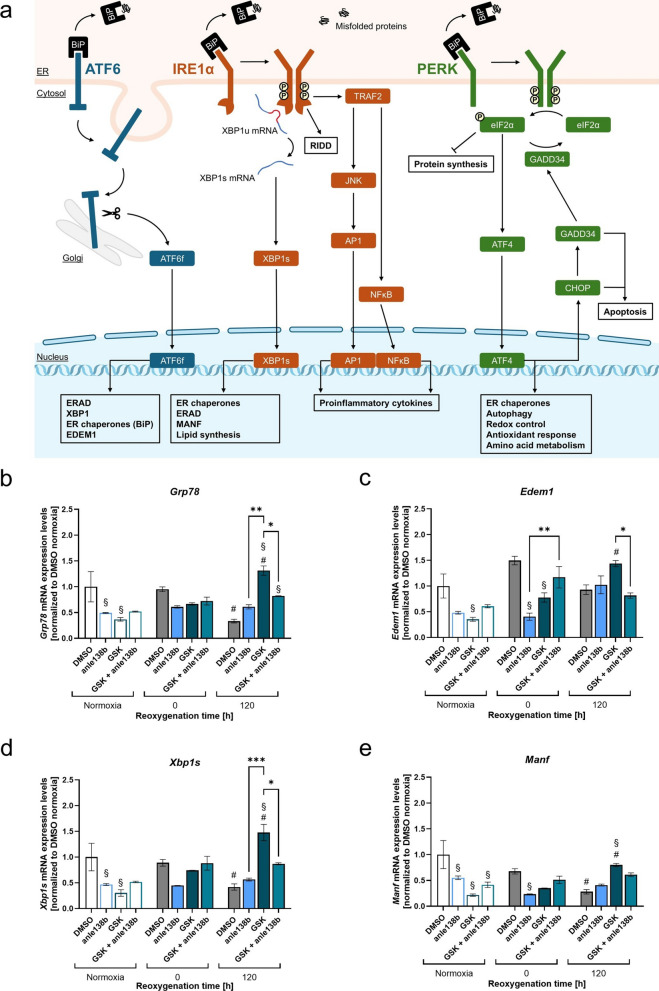
Post-hypoxic mRNA levels of the ATF6 and IRE1α branch after anle138b or GSK2606414 treatment. **a** Schematic illustration of the ATF6, IRE1α and PERK pathway of the UPR and their downstream effects. **b–e** Graphs showing the post-hypoxic mRNA expression levels of markers of the ATF6 branch (*Grp78*, *Edem1*) and the IRE1α branch (*Xbp1s*, *Manf*) of the UPR in VN/VC flies after treatment with DMSO, anle138b, GSK or GSK + anle138b. Target genes are presented as ratios to the constitutive gene *Actin5c* and normalized to the control normoxia. Data are shown as means ± SEM of 3 independent experiments, including 3 technical replicates per genotype and experiment (total of 180 male flies for each treatment and condition). Multiple comparisons two-way ANOVA followed by Tukey post-hoc. # indicates significance compared to the corresponding normoxia *p* < 0.05. § indicates significance compared to the corresponding DMSO control *p* < 0.05. *p < 0.05, **p < 0.01, ***p < 0.001

We next analyzed the impact of anle138b and GSK on the activation of the post-hypoxic UPR. We assessed the expression levels of *Grp78*, *Edem1* (ATF6 branch), *Atf4*, *Gadd34*, *dPerk*, *eIF2α*, *Xrp1* (PERK branch), *Xpb1s* and *Manf* (IRE1α branch) throughout a reoxygenation period of 120 h (Fig. 7d, 12).

Hypoxia markedly activated the PERK arm with significantly elevated expression levels of *dPerk*, *Xrp1* and *Gadd34* in the DMSO group, peaking after 0 h of reoxygenation and returning to normoxic levels after 24 h of reoxygenation (*p* < 0.002, Fig. 7e–i).

Treatment with anle138b and/or GSK attenuated this post-hypoxic upregulation. Notably, the inhibitory effect of GSK appeared more pronounced under normoxic conditions, while, after hypoxia, anle138b was more effective at downregulating the UPR and particularly the PERK branch (*dPerk*, *eIF2α*, *Xrp1* and *Gadd34*, Fig. 7e–i). For example, the crucial proapoptotic PERK marker *Xrp1* was reduced significantly more after anle138b treatment compared to GSK or combined treatment after 0 h of reoxygenation (*p* = 0.03, Fig. 7h).

**Fig. 13:**
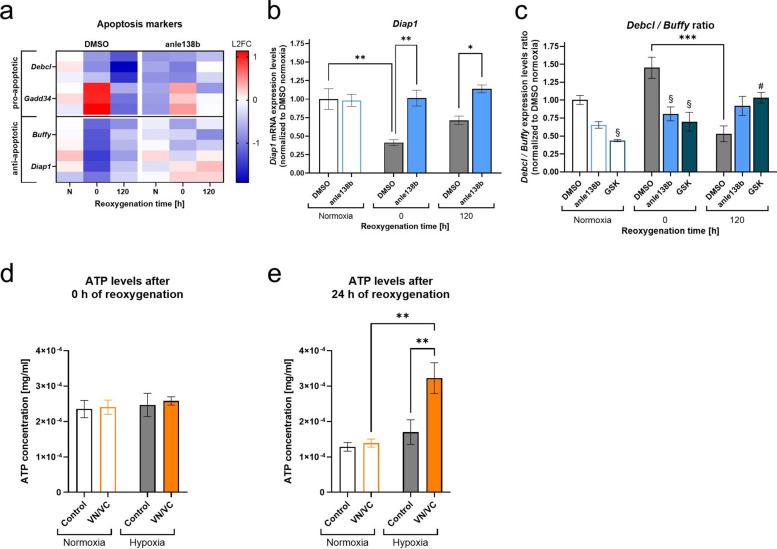
Post-hypoxic ATP levels and mRNA levels of proteins involved in apoptosis after treatment with DMSO, anle138b or GSK2606414. **a** Heatmap displaying the post-hypoxic mRNA levels of pro-apoptotic markers (*Debcl, Gadd34*) and anti-apoptotic markers (*Buffy*, *Diap1*) at different reoxygenation timepoints (0 and 120 h after hypoxia) after treatment with DMSO or anle138b prior to the hypoxia. Target genes are presented as L2FC to the constitutive genes *Actin5c* and *eEF1α2* and normalized to DMSO normoxia. **b** Post-hypoxic mRNA expression levels of *Diap1* in VN/VC flies after treatment with DMSO or anle138b. Target genes are presented as ratios to the constitutive gene *eEF1α2* and normalized to DMSO normoxia. **c** Ratio of mRNA expression levels of the pro-apoptotic *Debcl* and the anti-apoptotic marker *Buffy* in VN/VC flies after treatment with DMSO, anle138b or GSK2606414. Target genes were analyzed as ratios to the constitutive genes *Actin5c* and *eEF1α2* and normalized to DMSO normoxia. **d–e** ATP levels in VN/VC and control flies after 3 h of severe acute hypoxia following 0 h (**d**) and 24 h (**e**) of reoxygenation. Data are shown as means ± SEM of 3 independent experiments, including 3 technical replicates per genotype and experiment (total of 180 male flies for each treatment and condition). Multiple comparisons two-way ANOVA followed by Tukey post-hoc. # indicates significance compared to the corresponding normoxia *p* < 0.05. § indicates significance compared to the corresponding DMSO control *p* < 0.05. *p < 0.05, **p < 0.01, ***p < 0.001

To confirm that PERK upregulation following hypoxia-induced α-Syn aggregation promotes apoptosis, we analyzed the mRNA expression levels of Death-associated inhibitor of apoptosis 1 (*Diap1*), *Debcl* (*Drosophila* homolog of BAX) and *Buffy* (*Drosophila* homolog of Bcl-2, Fig. 13a). In the DMSO control group, *Diap1* was found significantly downregulated immediately after hypoxia (*p* = 0.0047), accompanied by a transient increase in the *Debcl/Buffy* ratio, peaking directly after hypoxia, indicating a shift towards apoptosis. Treatment with anle138b significantly stabilized *Diap1* expression levels over a 120 h reoxygenation period and markedly reduced the *Debcl/Buffy* ratio directly after hypoxia compared to DMSO control (*p* < 0.0424, Fig. 13b–c). Notably, PERK inhibition via GSK treatment reduced the *Debcl/Buffy* ratio in a similar way to anle138b, suggesting that the PERK/Xrp1/GADD34 pathway plays a key role in α-Syn aggregation-mediated apoptosis during early post-hypoxic reoxygenation.

To assess early metabolic readouts of apoptosis, we measured post-hypoxic ATP levels after 0 h and 24 h of reoxygenation in *Drosophila melanogaster*. While no significant differences were observed immediately after hypoxia, ATP levels were significantly elevated in VN/VC flies compared to control flies and associated normoxia control after 24 h of reoxygenation (*p* < 0.0068, Fig. 13d–e). In conclusion, while PERK inhibitor GSK initially tended to increase α-Syn aggregation levels, aggregation inhibitor anle138b was able to effectively reduce post-hypoxic α-Syn aggregation at both reoxygenation time points. Treatment with anle138b solely had the strongest downregulating effect on the post-hypoxic UPR-response, in particular the PERK branch, and stabilized the expression of pro-apoptotic markers.

## Discussion

Post-stroke cognitive impairment is a major complication affecting more than half of stroke survivors worldwide every year [5, 6, 13, 56]. Cerebral hypoxia triggers a range of pathological mechanisms, including oxidative stress, mitochondrial impairment, autophagy imbalance, ER-stress, protein misfolding and α-Syn aggregation [14, 18, 19]. In this study we assessed the impact of different hypoxia paradigms (acute, repetitive, chronic) on α-Syn aggregation, neurobehavioral outcomes, and the post-hypoxic activation of the UPR in *Drosophila melanogaster*. Moreover, we tested the efficacy of small molecule aggregation inhibitor anle138b in mitigating these effects.

We observed that acute severe hypoxia (3 h, O_2_ < 0.3%) leads to higher levels of α-Syn aggregation than repetitive (5 × 1 h, O_2_ < 0.3%) and chronic (120 h, O_2_ between 9 and 11%) hypoxia. Post-hypoxic α-Syn aggregation elevated acute mortality rates, shortened the life expectancy and prolonged the post-hypoxic recovery time. In addition, it led to impaired decision-making and an upregulation of the proapoptotic PERK branch of the UPR. The aggregation inhibitor anle138b sufficiently counteracted these detrimental effects by reducing post-hypoxic α-Syn aggregation and downregulating the PERK arm of the UPR. Our findings provide evidence of a potential role of hypoxia-induced α-Syn aggregation in cognitive and neurobehavioral impairments after stroke and identify aggregation inhibitor anle138b as a potential therapeutic approach in its prevention and treatment.

The brain relies on a continuous supply of oxygen. However, certain pathological conditions can cause hypoxic states in the brain. Cerebral hypoxia can manifest in various forms and durations. Acute hypoxia may result from a sudden cessation of the blood flow, leading to generalized (e.g., cardiac arrest) or focal hypoxia (e.g., ischemic stroke). Vascular pathologies or obstructive sleep apnea syndrome can lead to an intermittent reduction of the oxygen supply, triggering repetitive or chronic hypoxic states. Moreover, conditions such as anemia or chronic respiratory disorders (e.g., COPD) can lead to chronic oxygen deprivation in the brain. Recent studies suggest that ischemia/hypoxia increases α-Syn expression and aggregation in the brain [5, 20, 21, 57]. However, it has not yet been investigated whether the type of hypoxia influences α-Syn aggregation. Therefore, we compared α-Syn aggregation levels after acute, repetitive and chronic hypoxia in *Drosophila melanogaster*. We conducted the experiments in our very effective self-designed hypoxia chamber to guarantee constant air pressure, humidity, temperature and oxygen levels < 0.3% O_2_, as we have previously demonstrated that varying environmental factors during hypoxia may affect the reproducibility of the results [18, 44, 45]. We found that acute severe hypoxia resulted in significantly higher α-Syn aggregation levels than repetitive or chronic hypoxia (Fig. 1). It is known that hypoxic preconditioning can promote resilience against hypoxic and oxidative stress [58–63]. Chronic or repetitive exposure to hypoxic environments may also trigger adaptive mechanisms that reduce α-Syn aggregation, emphasizing the complexity of the mechanisms promoting and preventing hypoxia-induced α-Syn aggregation.

This raises another important question: is α-Syn aggregation triggered primarily by hypoxia itself or by reoxygenation? Lohmann et al. (2022) observed rising levels of aggregated α-Syn in mouse brains between 14 and 360 days post-stroke, Kim et al. (2016) and Wang et al. (2020) detected elevated aggregation levels 24 h post-stroke in their rodent models. However, none of the abovementioned studies measured aggregation levels directly after ischemia/hypoxia to differentiate between aggregation occurring during hypoxia and during reoxygenation [5, 20, 57]. Focusing on acute severe hypoxia, we found that aggregation levels were already significantly increased directly after the hypoxia compared to normoxia control in *Drosophila melanogaster* (Fig. 1). This indicates that a considerable amount of aggregation is already induced during hypoxia. In contrast to our in vivo model, the HEK-293 cells presented no difference in α-Syn aggregation directly after hypoxia, but aggregation levels gradually increased during reoxygenation (Fig. 2). One reason could be that neurons are more susceptible to α-Syn aggregation or more vulnerable to hypoxia than HEK-293 cells [64, 65]. Moreover, cellular and molecular interactions in a multi-organ species might contribute to a different α-Syn aggregation pattern compared to the monocellular HEK-293 cell model. We also found that during the reoxygenation period in HEK-293 cells, there was a shift in α-Syn aggregation from medium to high levels, while the number of cells with low aggregation levels remained constant (Fig. 2). This suggests a possible threshold level for α-Syn aggregation, below which cells can tolerate the aggregation that occurs. Only when this threshold is exceeded, adaptive mechanisms may become overwhelmed, causing α-Syn aggregation to escalate over time.

We come to the conclusion that hypoxia causes α-Syn aggregation, which is further exacerbated during reoxygenation when adaptive mechanisms in the cell are unable to cope with the accumulating aggregates.

We observed markedly elevated post-hypoxic α-Syn aggregation levels over a time period of 10 days in *Drosophila melanogaster*, indicating a long-term persistence of α-Syn aggregates. However, long-term measurements of α-Syn aggregation are subject to certain limitations that preclude a reliable analysis beyond the 10-day reoxygenation period in our model: First, sporadic α-Syn aggregation may occur independently of the initial hypoxic insult over time. We observed that even under normoxic conditions, α-Syn aggregation levels increased over time. This limits the interpretation of later aggregation assessments as being specifically attributable to the initial hypoxic stimulus. Second, long-term aggregation measurements in in vivo systems are subject to selection bias. Since α-Syn aggregation contributes to increased post-hypoxic mortality, only the more resilient individuals survive to later time points. Consequently, flies with the highest α-Syn burden die early, leading to an underestimation of aggregation levels in the hypoxia-surviving cohort compared to the normoxia control. To detect α-Syn aggregation, we utilized a BiFC system called α-Syn:VENUS. BiFC models have been used in multiple studies since 2008 to monitor α-Syn oligomerization and aggregation [50, 66–70]. However, Frey et al. demonstrated that there are certain limitations to the usage of α-Syn:VENUS, as unequal expression levels of both BiFC-fragments might impair its ability to detect aggregation and the modification of the α-Syn protein might influence its tendency to form aggregates, potentially leading to an over- or underestimation of aggregation levels [71]. Multiple studies have found that elevation in VENUS fluorescence levels was highly indicative of α-Syn oligomerization [66, 70, 72]. We performed a Thioflavin T assay in *Drosophila melanogaster* head lysates and utilized Thioflavin S staining in HEK-293 cells to validate that the VENUS signal reflects aggregate formation. We observed that VENUS and Thioflavin fluorescence intensities correlated very strongly across samples. Our data indicate that VENUS is a very sensitive tool to detect early oligomerization, but also the formation of large aggregates. In this study, we focused on comparing relative effects between experimental and control conditions to ensure that the observed inter-group differences were driven by α-Syn aggregation. Despite the abovementioned limitations, BiFC remains a valuable and well-established tool to detect α-Syn oligomers, as it is highly sensitive for α-Syn oligomerization and allows for a simple and reproducible quantification of α-Syn aggregation levels [73, 74].

Hypoxia-induced α-Syn aggregation might contribute to higher mortality rates and post-hypoxic motor deficits. Kim et al. (2016) reported that α-Syn knockout mice exhibited better post-hypoxic motor function and lower mortality rates in the first 7 days post-stroke compared to control mice [20]. In line, we observed that *Drosophila melanogaster* expressing α-Syn presented significantly higher mortality rates than control flies after hypoxia. However, α-Syn aggregation did not affect the severity of motor impairment, as hypoxia similarly decreased the activity levels of both genotypes. One possible explanation is that motor deficits caused by α-Synucleinopathies might become detectable only in later stages after hypoxia. It is known that motor deficits occur relatively late in PD patients [75]. Nonetheless, we observed that the post-hypoxic recovery time was significantly prolonged in flies expressing α-Syn, which, in line with the increased mortality, indicates a detrimental effect of α-Syn aggregation on the acute post-hypoxic phase (Figs. 3, 4).

As α-Synucleinopathies are primarily associated with chronic pathologies, it is crucial to study possible long-term effects of hypoxia-induced α-Syn aggregation. The longest preclinical observation period was conducted by Lohmann et al. (2022), who monitored the development of motor deficits between 180 and 360 days after ischemia in a MCAO mouse model [5]. However, no study has yet investigated the effects of hypoxia-induced α-Syn aggregation on the overall life expectancy. Many rodent models used in past research have limitations in this regard, as their relatively long life expectancy prevents observation over their entire lifespan. In contrast, *Drosophila melanogaster*, with a life cycle of approximately 80 to 100 days, provide a unique advantage, allowing for a complete lifespan observation [76]. We found a significantly lower life expectancy in flies with α-Syn compared to control flies. To eliminate the possibility that the observed decrease in life expectancy was primarily due to their higher acute mortality rate, we analyzed the death incidence of the flies, excluding those that died within the first 48 h after hypoxia. We found that even after exclusion of these acute deaths, flies with α-Syn died earlier and in a shorter time span than the control flies (Fig. 3). This highlights the potential role of hypoxia-induced α-Syn aggregation on chronic long-term effects.

Our findings suggest that hypoxia-induced α-Syn aggregation severely impairs the acute outcome and recovery phase after hypoxia, but also negatively affects long-term parameters such as the overall life expectancy.

Neurodegeneration is a complex and heterogenous process that can be investigated through a range of different morphological and functional markers. In our study, we primarily focused on functional readouts of neurodegeneration such as cognition, sleep-cycle activity and locomotor function. We found that hypoxia-induced α-Syn aggregation leads to altered sleeping patterns, prolonged locomotor recovery and significantly impaired decision-making (Figs. 4, 6). Assessment of morphological alterations could further validate our findings, but these analyses (e.g. pseudopupil quantification or assessment of neuromuscular junctions) are more suitable for assessing gene-specific neurotoxicity in genetic reporter lines and are less applicable to adult flies or longitudinal investigation of chronic neurodegeneration. We specifically chose readouts that mimic the clinical phenotype of post-stroke cognitive impairment and that align with typical manifestations of synucleinopathies such as Lewy body dementia and Parkinson’s disease. In summary, our data provide evidence that hypoxia-induced α-Syn aggregation markedly impairs long-term locomotor function, neurobehavior and cognition, underlining its key pathological role in the development of post-hypoxic neurodegeneration.

In α-Synocleinopathy models and in post-mortem brain tissue analysis of patients with dementia with Lewy bodies, disruptions in the protein quality control and activation of the UPR appear to be key mechanisms [77, 78]. Unlike these chronic models of α-Syn aggregation, acute severe hypoxia is a distinct acute stressor that leads to rapid aggregation and potentially alters the UPR activation pattern. While recent studies have reported UPR activation after hypoxia/ischemia, the mechanistic link between hypoxia-induced α-Syn aggregation and the UPR has not yet been investigated [51, 79–82]. We used RT-qPCR to measure branch-specific UPR targets, which is a commonly used approach due to the transcriptional nature of the UPR. Although protein-level analysis would further validate the findings, it is technically challenging in *Drosophila melanogaster* due to limited availability of specific antibodies. Despite this limitation, we believe our RT-qPCR data provide meaningful insights into UPR activation in this model. We demonstrated that flies expressing α-Syn present a significantly stronger post-hypoxic upregulation of the PERK/Xrp1/GADD34 axis, whereas inhibition of α-Syn aggregation leads to a downregulation of several markers of PERK activation, indicating a pivotal role of α-Syn aggregation in the post-hypoxic PERK activation (Figs. 5, 7). PERK inhibition increases α-Syn aggregation levels in the acute stage after hypoxia (Fig. 7). This indicates that the PERK branch activation might be an initial cellular response to the rapidly accumulating α-Syn aggregates after hypoxia. PERK is known to phosphorylate eIF2α, which leads to a global reduction of protein synthesis to reduce folding stress on the ER and might thereby reduce the load of misfolded α-Syn, effectively reducing α-Syn aggregation in the initial phase [83]. In contrast, flies treated with PERK inhibitor GSK presented lower post-hypoxic aggregation levels after 120 h of reoxygenation compared to the DMSO vehicle control. This effect may be attributed to a reactive overactivation of the PERK branch after the inhibitory effect of GSK subsides. Note, that we treated the flies only 24 h prior to the hypoxia and GSK levels might have been below therapeutic dose after 120 h of reoxygenation. In line, we observed a significant upregulation of PERK markers, including *eIF2α*, in GSK-treated flies 120 h after the hypoxia.

However, under sustained ER-stress, the PERK branch is known to activate a cascade of proapoptotic pathways, causing neuronal cell death [84]. We assessed the expression levels of several markers involved in the regulation of apoptosis and found that α-Syn aggregation-dependent PERK activation caused an increase in the *Debcl/Buffy* mRNA ratio and an acute downregulation of anti-apoptotic *Diap1*, indicating a shift towards apoptosis. Anle138b treatment stabilized the expression levels of apoptosis-associated markers, mitigating acute post-hypoxic PERK-dependent apoptosis (Fig. 13). While there is evidence that inhibiting the PERK pathway is beneficial in PD and other neurodegeneration models, its role in the adaptation to hypoxia is controversially discussed [48, 85]. Some studies found reduced neuronal death and improved motor function in rat models (MCAO, SAH) after treatment with PERK inhibitor GSK [86, 87]. Other studies observed increased cell death and decreased hypoxia tolerance after GSK treatment or PERK knockout in cell culture models [88–90]. The timepoint of administration of GSK or PERK-inhibition, specifically whether during hypoxia or post-hypoxia, might be crucial for the outcome. Most studies reporting worsened outcomes after PERK inhibition used either a knockout model or administered GSK before hypoxia, while those showing protective effects administered GSK during reoxygenation. This underlines the dual function of the PERK branch, playing a crucial role in the initial response to the accumulating α-Syn aggregation during hypoxia, but also promoting proapoptotic pathways when prolonged ER-stress exceeds coping capacity, leading to neuronal cell death as well as neurological and cognitive impairment.

Because of the dual role of the PERK branch, its direct manipulation with drugs like GSK may be problematic as a therapeutic approach. Depending on the timing and the stage of stress it may produce adverse effects and fail to effectively target cognitive and neurological impairment after stroke. We propose that inhibiting α-Syn aggregation is a more promising strategy to combat neurological and cognitive dysfunction after stroke, as we have identified hypoxia-induced α-Syn aggregation as an important contributor to the post-hypoxic activation of the PERK branch.

The aggregation inhibitor anle138b has demonstrated significant therapeutic potential in several synucleinopathy models of PD and multiple system atrophy, displaying not only effective reduction of α-Syn oligomerization but also a favorable biodistribution and successful penetration of the blood–brain barrier [26, 29, 30, 33, 34]. However, its biodistribution properties in *Drosophila melanogaster*, particularly under hypoxic conditions, and its impact on hypoxia-induced α-Syn aggregation have not yet been investigated. We demonstrated that radiolabeled [^131^I]I-anle138b accumulates in the brains of *Drosophila melanogaster*, with hypoxia further enhancing this uptake (Fig. 6). As *Drosophila melanogaster* lack a conventional blood–brain barrier, these findings imply that post-hypoxic cellular mechanisms may contribute to the increased uptake of anle138b.

Anle138b treatment reduced post-hypoxic α-Syn aggregation levels and mitigated the activation of the PERK branch. The flies’ decision-making ability was vastly improved after treatment with anle138b 10 days after hypoxia. While anle138b treatment did not change the acute post-hypoxic mortality rate, the life expectancy was significantly improved (Fig. 6). This finding might be linked to the improved cognitive status we observed in the anle138b-treated group. Cognitive impairment may lead to disrupted eating and drinking behavior as well as orientation issues, ultimately resulting in increased frailty. Noteworthy, all of the abovementioned beneficial effects of anle138b were observed despite the relatively short treatment period of just 1 to 5 days prior to hypoxia, highlighting the critical pathological role of hypoxia-induced α-Syn aggregation in the post-hypoxic period. Furthermore, single treatment with anle138b directly after hypoxia was similarly able to dampen the rapid accumulation of α-Syn aggregates in HEK-293 cells during early reoxygenation (Fig. 11). Our findings suggest that anle138b is a promising therapeutic agent for the prevention and treatment of PSCI.

In conclusion, this study provides evidence that acute severe hypoxia causes higher levels of α-Syn aggregation compared to chronic or repetitive hypoxia, indicating that chronic hypoxic states may initiate adaptive mechanisms, which are insufficient to cope with the acute stress of severe hypoxia. Hypoxia-induced α-Syn aggregation leads to increased acute mortality rates, reduced life expectancy and longer recovery times. The PERK branch of the UPR is activated in response to the post-hypoxic accumulation of α-Syn aggregates. While initially inducing protective mechanisms to decrease ER folding stress, it ultimately causes the expression of proapoptotic proteins such as CHOP (Xrp1 in *Drosophila melanogaster*) and GADD34, potentially contributing to neuronal cell death and subsequent cognitive decline. The aggregation inhibitor anle138b exhibits sufficient brain uptake under hypoxia and effectively reduces hypoxia-induced α-Syn aggregation, thus mitigating PERK activation and leading to improved life expectancy and cognitive function. This study strongly supports further investigation of anle138b as a promising therapeutic approach for the prevention and treatment of PSCI.

## Appendix

See Figs. 8, 9, 10, 11, 12 and 13.

## Abbreviations

ATF4: Activating transcription factor 4
ATF6: Activating transcription factor 6
BiFC: Bimolecular fluorescence complementation
CHOP: C/EBP-homologous protein
DAM: Drosophila activity monitoring
DMEM: Dulbecco’s Modified Eagle Medium
DMSO: Dimethyl sulfoxide
EDEM1: ER degradation enhancing alpha-mannosidase like protein 1
eIF2α: Eukaryotic translation initiation factor 2α
ER: Endoplasmic reticulum
ERAD: ER-associated degradation
FBS: Fetal bovine serum
GADD34: Growth arrest and damage-inducible 34
GRP78: Glucose-regulated protein 78
GSK: GSK2606414
HEK-293 cells: Human embryonic kidney 293T cells
IRE1α: Inositol-requiring enzyme 1α
MANF: Mesencephalic astrocyte-derived neurotrophic factor
MCAO: Middle cerebral artery occlusion
PERK: Protein kinase RNA-like endoplasmic reticulum kinase
PSCI: Post-stroke cognitive impairment
Radio-TLC: Radio Thin Layer Chromatography
RCP: Radiochemical purity
RIDD: Regulated IRE1-dependent decay
RIPA: Radioimmunoprecipitation assay
SAH: Subarachnoid hemorrhage
UPR: Unfolded protein response
VBFD: Value-based feeding decision
XBP1s: X-box binding protein 1
α-Syn: α-Synuclein

## Declarations

### Availability of data and materials

All data supporting the findings of this study are available within the article and its supplementary information files. Raw data and additional information related to this paper may be requested from the corresponding author upon reasonable request.
